# Understanding Identity Changes in Psychosis: A Systematic Review and Narrative Synthesis

**DOI:** 10.1093/schbul/sbaa124

**Published:** 2020-09-29

**Authors:** Maev Conneely, Philip McNamee, Veenu Gupta, John Richardson, Stefan Priebe, Janelle M Jones, Domenico Giacco

**Affiliations:** 1 Unit for Social and Community Psychiatry, WHO Collaboration Centre, Queen Mary University of London, London, UK; 2 Department of Primary Care and Mental health, Institute of Population Health, University of Liverpool, Liverpool, UK; 3 Department of Biological and Experimental Psychology, School of Biological and Chemical Sciences, Queen Mary University of London, London, UK; 4 Division of Health Sciences, Warwick Medical School, University of Warwick, Coventry, UK

**Keywords:** identity, self, schizophrenia, psychosis, self-experience, self-disorder

## Abstract

**Background and Objective:**

Experiencing psychosis can be associated with changes in how people see themselves as individuals and in relation to others (ie, changes in their identity). However, identity changes receive little attention in treatment, possibly due to a lack of clarity or consensus around what identity change means in people with psychosis. We aimed to create a conceptual framework synthesizing how identity changes are understood in the psychosis literature.

**Methods:**

Electronic databases were searched up to April 2020. Studies about identity changes among people with psychotic disorders were analyzed using narrative synthesis by a collaborative review team, including researchers from different disciplines, clinicians, and people who have experienced psychosis.

**Results:**

Of 10 389 studies screened, 59 were eligible. Identity changes are understood in 5 ways as (1) characteristics of psychosis, (2) consequences of altered cognitive functioning, (3) consequences of internalized stigma, (4) consequences of lost roles and relationships, and (5) reflections of personal growth. These 5 understandings are not mutually exclusive. Across a heterogeneous literature, identity changes were mostly framed in terms of loss.

**Conclusions:**

Our conceptual framework, comprising 5 understandings, highlights the complexity of studying identity changes and suggests important implications for practice and research. For clinicians, this framework can inform new therapeutic approaches where the experience and impact of identity changes are acknowledged and addressed as part of treatment. For researchers, the conceptual framework offers a way of locating their understandings of identity changes when undertaking research in this area.

## Introduction

People’s sense of identity, or their sense of who they are as individuals and in relation to others, can change throughout their lives. Important transitions in life can be catalysts for identity change. Leaving secondary school to study at university,^1^ retirement from work,^2,3^ brain injury,^4^ recovering from a stroke,^5^ and becoming a parent^6^ are events where individuals may question who they were, who they now are, and who they might become.

Many people describe experiencing symptoms of psychosis, as well as being diagnosed with a psychotic disorder, as having a profound impact on their lives and sense of identity.^7–10^ Traditional psychiatric models describe altered or disordered self-experience as experiences involving a “deformed sense of first-person perspective.” ^11^ These experiences of identity change are thought to be useful in identifying psychosis, with some researchers arguing that schizophrenia should be regarded as a disorder of self-experience.^12,13^ The recovery approach does not share this view, but sees identity as an essential channel to recovery, which involves “rebuilding or redefining a positive sense of identity.” ^12,14–28^ These, and other approaches to studying identity changes, have been compared and contrasted in existing reviews.^23,27,29–31^ These different approaches, with their varying emphases and theoretical assumptions, have created division and limited knowledge sharing between research in the same broad area. To overcome these issues and move research forward, a conceptual framework is needed to bring together ways in which identity changes in psychosis are understood. 

The social identity approach (SIA), which originates from research in social psychology, may be useful in considering identity changes in psychosis. This approach, comprised of Social Identity Theory^32^ and Self-Categorization Theory,^33^ argues that an important part of how we see ourselves comes from our belonging to, and our identification with, social groups.^32,34,35^ This understanding of identity is fluid and dynamic: as context changes, the way people see themselves and others also shifts.^28^ Research in this approach has shown that strong group identifications confer mental health benefits by giving people a sense of belonging, meaning and social support.^36–40^ Critically, this research has shown that changes in people’s lives can promote changes in their sense of identity and, through this, affect their mental health.^1,4–6,39–50^

There is currently no standardized framework for understanding identity changes in psychosis, despite the evidence that many people diagnosed with psychosis experience identity changes and indications that identity influences mental health. Understanding how identity changes are described may help clinicians’ and patients’ efforts to manage and make sense of identity changes and the impact these have on patients’ lives. We aim to systematically identify and synthesize research on identity changes in psychosis to create a conceptual framework of how identity changes are understood in the psychosis literature. The conceptual framework will be discussed using research from the SIA. This will further reduce barriers between disciplines and allow the understandings of identity changes in the psychosis literature to be considered within the larger body of evidence from social psychology.

## Methods

A systematic review and narrative synthesis was conducted following the protocol developed according to the PRISMA guidelines^51^ and registered on PROSPERO (CRD42018058965). 

### Worldview

This research was carried out within a pragmatic participatory worldview. Rather than being exclusively positivist or constructivist, the pragmatic approach allowed multiple perspectives on reality to be used based on how helpful they are in addressing the research aim.^52^ Participatory methods were equally influential in this project. People with experience of psychosis (V.G. and J.R.) and people with experience of providing psychiatry treatment (D.G. and S.P.) were involved, following existing guidance.^53,54^ The pragmatic participatory worldview influenced the protocol design, choices of method, coauthors, advisory expert panels, and the choice of theoretical lens used to discuss the results.

### Search Strategy

The search strategy spanned theoretical orientations and methods of studying identity changes by using a variety of data sources and terms to describe the sample of interest (ie, people diagnosed with a psychotic disorder) and the phenomenon of interest (ie, identity change). Search terms included: “identity,” “self,” and “narrative” combined with words to describe the population such as “psychosis” and “voice hearer.” PubMed, PsycINFO, Web of Science, and CINAHL were searched from inception until April 9, 2020. Gray literature (OpenGrey, GoogleScholar, and the British Library Catalogue) and reference lists of included studies were also searched (see supplementary material 1 for full search strategy).

### Screening and Selection Criteria

We included qualitative and quantitative research that: (1) sampled adults (at least *n* = 3), where at least 50% of the participants were diagnosed with a psychotic disorder (F20-F29 in the International Classification of Diseases,^55^ or equivalent) and (2) outlined a description (or model, framework, themes) of identity changes, broadly described as modifications in the social positioning of the self and other.^24^

Studies were excluded if identity changes were: (1) exclusively understood as a trait or personality based (eg, agreeableness or extroversion), (2) not assessed as *change* (ie, identity measured at one time point only), or (3) studied exclusively via observers (eg, description from family members, researchers, or clinicians). Articles were also excluded if they were books, chapters, dissertations, theses, forensic samples, reviews, or opinion pieces. Reviews were excluded but their references were searched.

Eligibility was assessed by M.C. and P.M. using predefined criteria published on PROSPERO. After duplicates were removed, a random sample of 20% of the identified texts was independently assessed for eligibility against the inclusion criteria by P.M. and M.C. Acceptable concordance was predefined (90% agreement); 92% was achieved. Disagreements were resolved by consulting J.M.J. and D.G.

### Data Extraction

Data were extracted independently by M.C. and P.M. on a sample of 10% of the included studies to trial the extraction sheet, which included study features, sample features (diagnoses and recruitment setting), and how identity changes were described. Quality assessment was not carried out as it is inappropriate when conducting an analysis of concepts.^56,57^

### Data Synthesis and Analysis

**Fig. 1.**
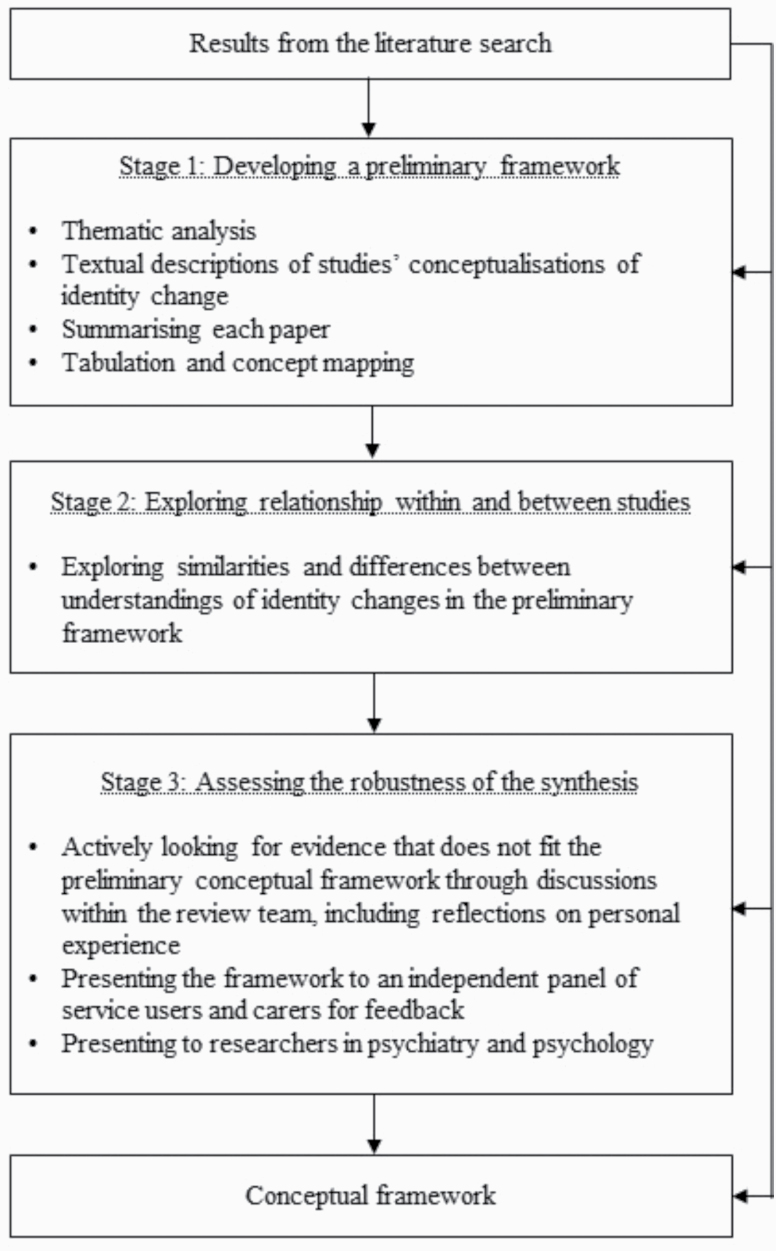
Modified narrative synthesis process.

To analyze the data, we modified the guidance on narrative synthesis proposed by Popay and colleagues^58^ to include 3 rather than 4 stages: creating a preliminary synthesis, exploring relationships within and between studies, and assessing the robustness of the synthesis (figure 1). The first stage was omitted as it only applies to synthesizing interventions. We combined our narrative synthesis with elements of conceptual framework analysis.^18,59^ Specifically, we drew on the guidance on managing sources of data, deconstructing concepts, and used techniques to integrate the synthesis into a conceptual framework, defined as “a network or a ‘plane’ of linked concepts.” ^59^ After carrying out an in-depth, inductive thematic analysis of the data extracted from the studies and mapping common concepts across studies, a conceptual framework was created and gradually refined (see supplementary material 2). Throughout the analysis and development of the conceptual framework of identity change, emerging ideas were discussed among the team. All authors were involved in refining the conceptual framework and describing the understandings of identity changes. In line with our pragmatic participatory worldview, 2 coauthors with lived experience of psychosis (V.G. and J.R.) documented their thoughts on the emergent conceptual framework, including how each understanding of identity change related to their personal experience of psychosis. These were collated and their reflections on the final framework are presented as part of the results. We used the SIA as a lens to discuss the findings.

## Results

**Fig. 2.**
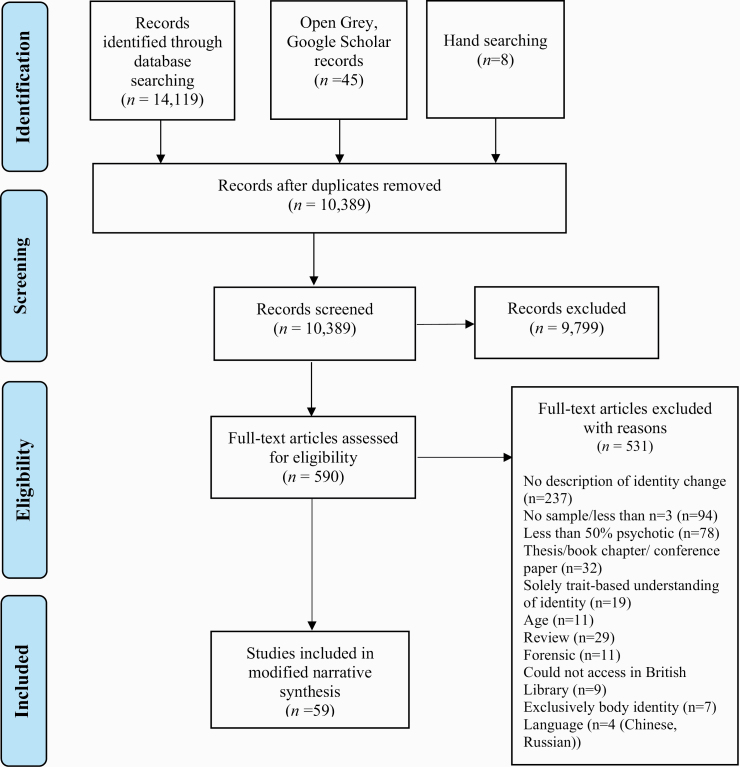
Flowchart of included studies.

Of the 10 389 publications screened, there were 59 eligible studies (figure 2). The studies were published between 1961 and 2020 and conducted in 14 countries, primarily in Europe (*n* = 28) and North America (the United States, *n =* 15; Canada, *n* = 9), and the remaining (*n* = 7) from other regions. Articles were written in 3 languages: English (*n* = 56), French (*n* = 2), and Spanish (*n* = 1). (The studies were extracted and translated into English by a native speaker and checked by a second native speaker.) Some studies explored the experience of identity changes and the meaning of identity changes to patients,^60^ while other studies explored the processes involved in identity changes (ie, its determinants and/or consequences),^61^ or combined both approaches.^62^ There were 36 distinct operationalizations of identity, with few studies using the same theoretical understanding of identity change or sharing the same way of assessing identity change (all included studies can be found in table 3 in supplementary material 3). 

**Fig. 3.**
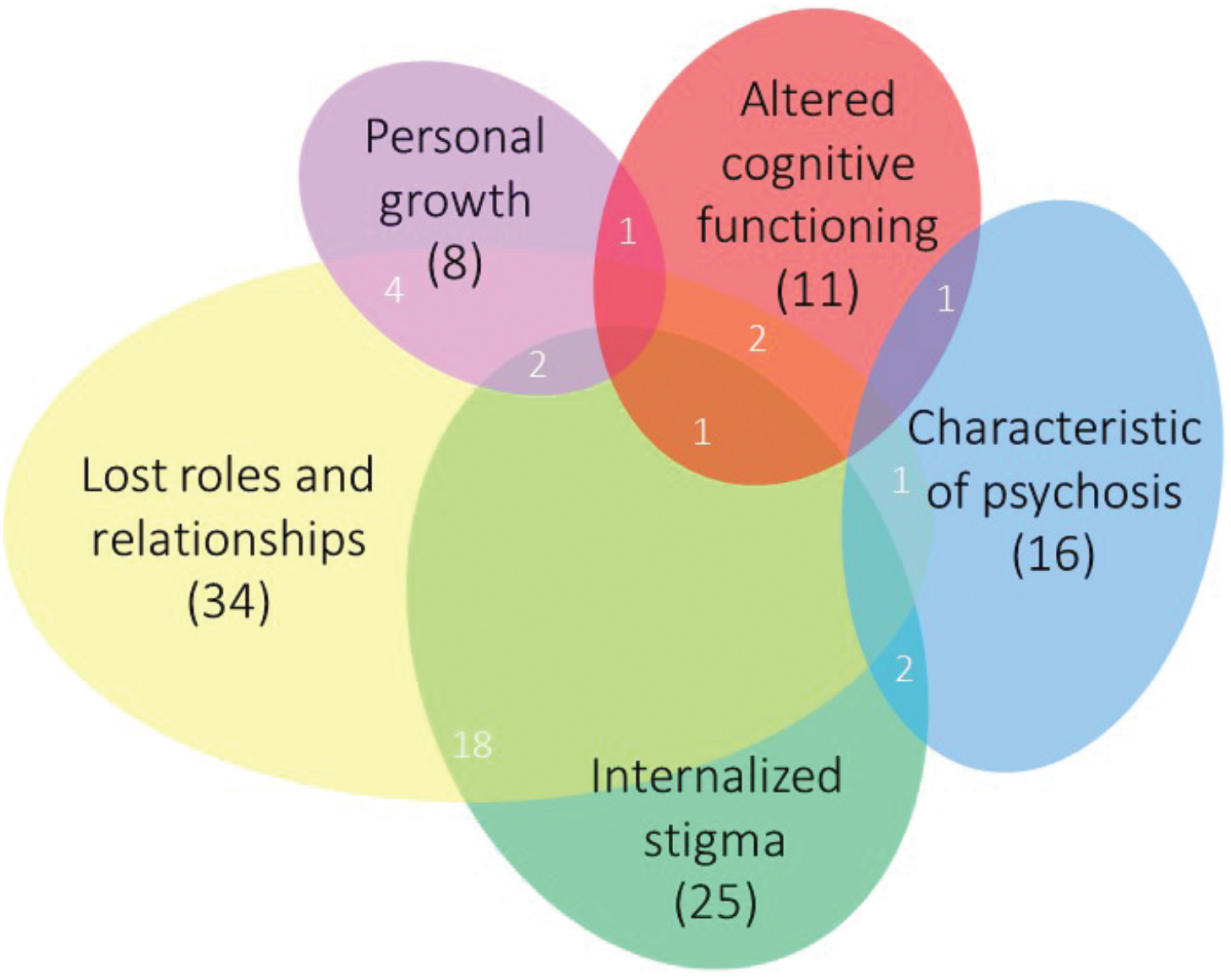
Visual representation of the conceptual framework of identity changes in psychosis, showing interrelationships between the 5 understandings.

The conceptual framework comprises 5 ways of understanding identity changes (figure 3). Identity changes are understood as: (1) characteristics of psychosis, (2) consequences of altered cognitive functioning, (3) consequences of internalized stigma, (4) consequences of lost roles and relationships, and (5) reflections of personal growth. Several studies described identity changes using more than 1 of the 5 understandings in the framework (figure 3; table 3 in supplementary material 3). The authors with experience of psychosis echo this complexity, describing having experienced and made sense of identity changes in different ways (table 2).

**Table 2.** Reflections on the Conceptual Framework

Overarching Reflections
The understandings of identity change we identified in the literature replicate my experience of psychosis to some extent, and how my self-concept has changed over the course of my life since experiencing this. Some of these understandings I relate to more than others—eg, the more negative ones such as “characteristics of psychosis” do not speak to me as much as others. V.G. These understandings are useful because they invite you to think. Some I find more relatable than others. Nevertheless, they all help me to consider how I, and others with similar experiences, might relate to the world, opening up some potential for healing. Loss of identity always being described as negative is surprising. Maybe the loss of identity is not all bad. Loss is a part of life and a part of change. For me, the understandings can be ordered in how well they relate to my experience of identity change in the opposite order to how they appear in the text, with the fifth (personal growth) fitting best and the first (characteristics of psychosis) fitting the least well with my experience. J.R.
1.Characteristics of psychosis
Neither of us found this way of understanding identity change related to our experience, primarily because it seemed like the “problem” was located within the individual. This way of understanding identity change as self-disorder seems pathologizing. I think it does to some extent reflect my experiences, but the language of “anomalous” and “self-disorder” is very negative. Some of the descriptions do apply to how I experienced psychosis: when I was consumed by psychosis my whole reality was changed, which included how I saw myself. V.G. I am cynical of this way of understanding identity change. How can anyone objectively know or observe what normal self-experience is? It makes me uncomfortable that the change in identity can only be bad—in this case, not only bad but also a sign of “disorder.” That being said, there are aspects of the description of “self-disorder” that do speak to me. When I was in the psychosis, I felt merged and profoundly at one with the world and at the same time felt extremely alienated from the people around me. J.R.
**2.**Consequence of altered cognitive functioning
We had different reactions to this understanding of identity change. This understanding made sense to me. Looking back, there was no clarity in my cognitions at the time, which affected how I saw myself, and medication helped to bring clarity back into my thinking and bring me back to myself. Although I think it captures something I experienced, I also think this understanding is a bit negative because it focuses on loss. V.G. I’m skeptical about the implied worldview in this understanding. I think not considering the environment loses sight of the real context that cognitions happen in, and also that research happens in. People currently in the psychosis might not see performing on a cognitive test as important. I think understanding changes in how you see yourself as related simply to cognitive ability is reductive, paternalistic, and does not have helpful or positive implications for how I see myself. J.R.
**3.**Consequence of internalized stigma
We have both definitely seen the influence of stigma and internalized stigma, on our identities, and still see the impact it has on us. I think other people’s perceptions color the way you think about yourself and this makes you feel different and inferior somehow. It was important for me to hold other identities, such as being a mental health professional and now a researcher, to think of myself in a more positive way. This way of understanding identity change also shows how the environment and other people really influence how you think about yourself and takes into account social inequalities and the environmental context that psychosis happens in, which, in my experience, is more accurate. I don’t think you can really separate the way you see yourself from how other people see you, so of course, the stigma attached to psychosis plays an important part in shaping my identity. V.G. The construct of internalized stigma shifts the blame to the person and away from the society it is created in. Discrimination happens, whether overt and obvious or more covert, and that affects how you relate to others and yourself. It changes the way I behave. I am over-aware of how I might be coming across: I work too much or arrive places too early, I err toward apathy so my enthusiasm isn’t mistaken for mania. This awareness and attempt at managing how I appear to others is the result of stigma, and working to avoid fitting a stereotype, and to this day, it affects how I see myself and my behavior. J.R.
**4.**Consequence of lost roles and relationships
We have both felt that roles are important shapers of our identity—both losing and gaining relationships, whether this was related to psychosis or other experiences, make us who we are. Having worked as an expert by experience for clinical psychology programs, I have come to identify with my diagnosis of psychosis due to the value this experience holds in educating trainee psychologists. My identity as a person with psychosis has provided me with a new role in life and has changed my self-concept positively, through feeling accepted within this role—although this was not the case when I was first diagnosed, in large part because of the stigma attached to the diagnosis. Through these roles, I came to see the identity of being a person with psychosis as positive and identified with it more over time. V.G. I’ve felt a type of grief carried in the deaths of relationships and roles, in the possibilities that are lost when these connections disappear. How you relate to others makes up a lot of how you view yourself. I’ve lost certain roles and relationships, and others have been strengthened and formed because of my identity as a mad person, which is a positive identity for me now. J.R.
**5.**Reflection of personal growth
This way of understanding identity change made the most sense to us and we were surprised at how relatively uncommon it was in the literature. Understanding identity change as personal growth is my favorite way of conceptualizing identity change in psychosis that we found in the literature. I think that my unprocessed past manifested itself and resurfaced in the form of psychosis and this needed to happen for me to deal with the roots of the trauma I had experienced. The psychosis was almost a reflection of what I had previously experienced and I needed to face this to deal with the root cause. I agree that this needed to happen was inevitable and unavoidable. The change I experienced in my identity was an addition, not a loss, although it was distressing. The experience needed to happen to separate myself from psychosis and get myself back to myself. V.G. This is the understanding I found most alluring. I have always felt the psychosis was inevitable, like a volcano exploding or my life vomiting itself up. The experience answered a lot of questions I had about my identity in life, took me to the limits of thought deviancy, and gave me a humility in how I look at reality. If I give in to the full romanticism of it, I like to think it made me a better human, as it put me in touch with my own fragility. It was both painful and a type of growth. J.R.

### Identity Changes Are Characteristics of Psychosis

The first way of understanding identity changes is as a personal or self-focused modification of how one experiences oneself as an individual. Here, identity change focuses on the self, and how a disruption in the self has an impact on how a person feels and behaves toward others. This change in self is conceptualized as a “self-disorder,” a deviation from normal functioning, which is determined by a trained psychiatrist applying a checklist, and/or in interviews with patients.^11,63–66^ The self is seen as “anomalous,” “pathological,” and/or “fragmented,” and changing how an individual relates to others.^67,68^ There are several types of self-disorders, including a “sense of being alienated from oneself and others,” “experiencing one’s self as merged with the world,” and experiencing important changes in “values and/or perception of the self.” ^69^ Self-disorder is described as a fundamental feature of psychotic disorders; it is most typical of schizophrenia and has been used to differentiate schizophrenia from other severe mental illnesses.^31,63,70^ Research suggests that self-disorder is a marker of vulnerability for psychosis,^71^ a predictor of suicidality,^72^ related to the duration of untreated psychosis,^73,74^ and associated with cognitive impairment as measured by neurophysiological techniques.^75^ The change from normal self-experience to self-disorder comes about via intraindividual processes, (ie, independent of the person’s social environment) and is considered to be on a continuum with symptoms of psychosis: the difference between self-disorder and other symptoms is in quantity or severity, not quality.

### Identity Changes Are Consequences of Altered Cognitive Functioning

The second way of understanding identity change is as a consequence of how people process and integrate cognitive information.^76,77^ To maintain a sense of identity, there is a need to bring together the different parts of how you see yourself, which involves accessing and integrating different cognitive processes.^78,79^ Based on research showing people with psychosis perform less well on certain cognitive tasks, this literature explores these changed cognitive functions as the explanation and reason for identity changes. Processes studied included reflective function^77^—an individual’s ability to reflect on their and others’ thinking, meta-cognition^80^—an individual’s ability to think about their thinking, autobiographical memory^81–83^—an individual’s memories of significant events in their life’s narrative, and executive function^83,84^—the cognitive processes necessary for planning, monitoring and switching between tasks. Allé and colleagues^84^ found that patients’ ability to make connections between their life’s narratives and self-attributes was impaired compared with people without a diagnosis of schizophrenia. Quantitative studies explored associations between cognitive functioning and identity change, with some studies finding support for their hypotheses,^79,85^ and others not finding any association between cognitive functioning and identity changes.^82,86^ Qualitative studies also described cognitive capacities as necessary for the construction of identity.^77,84,87^

### Identity Changes Are Consequences of Internalized Stigma

The third way of understanding identity change is as a consequence of the internalized stigma related to psychosis. Stigma can be attached to diagnosis, to treatment environments (eg, psychiatric hospitals), or to certain behaviors or circumstances related to diagnosis (eg, social withdrawal and unemployment).^88,89^ Stigma can be internalized such that individuals believe that they are somehow tainted as a result of their condition.^90^ The internalization of stigma is typically rooted in exposure to societal beliefs over the course of a person’s lifetime, rather than as a consequence of illness. This means that negative beliefs about psychosis are likely to have existed before the onset of illness, but these only have an impact on an individual’s identity when they have received a diagnosis.^66,91,92^

A specific conceptualization of the impact of stigma on identity is “illness identity” where “illness defines the totality of a person’s being, and restricts all aspects of their lives,” and in its extreme, “engulfment,” “whereby illness and its associated stigma entirely define self-concept.” ^93^ Being in hospital and taking medication were described as potential ways of increasing engulfment, as they act as symbols that patients are “entering a stigmatized class” both to themselves and to others.^94^

### Identity Changes Are Consequences of Lost Roles and Relationships

The fourth way of understanding identity change is as a consequence of the loss of roles and relationships in people’s lives. Changes in where individuals work or live due to being hospitalized may lead to the loss of status and purpose if they are unable to continue in their jobs, and separation or exclusion from relationships with friends, neighbors, colleagues, and family.^62,95–97^ Loss of these defining interactions can then promote identity change, especially when lost roles or relationships are not replaced with new ones. Corin^62^ suggests that “frequently hospitalized patients express a feeling of being ‘kept out of things’” at the interpersonal level. Roles and relationships have also been described as important in intentional, positive, identity changes. Connell explains: “The strengthening of relationships and return to familiar roles (e.g., work, study) and environments also helped to normalize the experience of self.” ^60^

### Identity Change Reflects Personal Growth

The fifth way of understanding identity change is as a reflection of personal growth. Here, psychosis is associated with a meaningful transformation in an individual’s sense of self and is understood as an evolution brought on by an event or experience in their lives. Identity change is not described as a negative, or as a loss, but rather is perceived as neutral or positive: the changed identity is a difference, rather than a deficit. Both identity change and psychosis are described as meaningful or “meant to happen.” In interviews about recovery and changes in self, one participant said: “I guess all the bottled up trauma sort of came out. (…) I think it was meant to happen. I think I needed to go through that to get better.” Another participant in the same study said “I think this happened for a reason, probably a good reason. (…) It gave me more self-knowledge I guess.” ^60^ Jarosinski^98^ also presented identity changes as a gain rather than loss, noting that “Almost all participants verbalized that the experience, good or bad, made them more human.”  

#### Interrelationships Between Understandings

The 5 understandings in the framework are not mutually exclusive (overlaps in figure 3; supplementary material 3). Articles that described identity changes in 2 or 3 different ways often explained how these processes might interact. People might see identity change as primarily the result of altered cognitive functioning, ie, exacerbated by internalized stigma^79^. Changed cognitive functioning might also lead to the loss of roles and relationships, by changing how a person interacts with others.^81,85^ Articles focusing on one way of understanding identity changes also reflected that other forms of identity change may co-occur or interact with one another. Identity changes understood as characteristics of psychotic disorders were often considered alongside, or as a precursor of, other understandings of identity changes. A disordered self can not only promote identity change directly but it might also lead to identity changes by altering cognitive functioning,^78^ and increasing internalized stigma,^67^ or the loss of roles and relationships.^97^ Stigma was a factor that many studies considered as also having an impact on identity, alongside one of the other understandings.^61,62,79,98–100^ There was no overlap between studies conceptualizing identity change as both a characteristic of psychosis and a form of personal growth, although personal growth did co-occur with other understandings in the framework.^60,101,102^ Although the 5 understandings are not mutually exclusive, the small numbers in the overlaps in figure 3 show that most of the research considers identity changes in a way that fits into a single understanding within the conceptual framework (with the exception of internalized stigma, which is often considered alongside other understandings). 

#### Commonalities and Differences

##### Loss

Across 4 of the 5 understandings, identity changes were described as negative, usually framed as a loss. This loss was described in indirect terms: identity change happens because of a loss of roles and relationships or loss of cognitive functioning following the onset of psychosis. Loss of identity, or “diminished self-experience,” was also described in direct terms as a part of, or a characteristic of, psychosis. Studies also described loss in more abstract terms, such as losing a positive sense of self. Understanding identity changes as the result of internalized stigma reflects this type of loss. Many articles did not explicitly refer to how loss came about, but the distressing symptoms of psychosis, and/or diagnosis were consistently seen as a pivot point between different versions of themselves.^100^ Many studies (*n* = 43) also described the possibility of identity gain and positive identity changes. However, the initial change in identity was generally considered to be a loss. Studies that described identity change as personal growth (*n* = 8) were the only ones that included a description of the initial identity change as neutral or positive.

##### Intrapersonal and Interpersonal Factors

The 5 understandings of identity change focus on identity changes in either primarily intrapersonal (characteristics of psychosis, altered cognitive functioning, and personal growth) or primarily interpersonal terms (roles and relationships). Internalized stigma is the only understanding of identity change that involves change on both an interpersonal level (becoming part of a stigmatized group) and an intraindividual level (the person internalizing stigma and applying it to themselves) (table 1). The ticks in table 1 show the features that are common to different understandings in the conceptual framework.

**Table 1.** Overview and Comparison Between Understandings

	Characteristics of Psychosis	Altered Cognitive Functioning	Internalized Stigma	Lost Roles and Relationships	Personal Growth
Identity change is a loss	✓	✓	✓	✓	
Changes in identity can happen only after diagnosis			✓		
Primarily intrapersonal factors implicated in identity change	✓	✓	✓		✓
Primarily interpersonal (social) factors implicated in identity change			✓	✓	

#### Reflections From Authors With Lived Experience

Two members of the review team documented their thoughts on the conceptual framework, based on their experiences of psychosis (V.G. and J.R.) (table 2).

## Discussion

There is no standardized way of understanding identity changes in psychosis, and because of this, people experiencing psychosis have few avenues to make sense of these changes. This review synthesized the available research on identity changes to create a conceptual framework. Identity changes in psychosis are understood as (1) characteristics of psychosis, (2) consequences of altered cognitive functioning, (3) consequences of internalized stigma, (4) consequences of lost roles and relationships, and (5) reflections of personal growth. In 4 of the 5 understandings in the conceptual framework, identity change is described as a loss. This framework provides a way of considering these alongside each other in a transtheoretical and pragmatic way.

### Strengths and Limitations

This is the first study to create a conceptual framework of identity changes in psychosis using a systematic approach. A particular strength is our focus on a predefined *concept* rather than on a word, meaning diverse approaches to the same subject matter could be considered together and included in the synthesis. As a result, the present synthesis cuts across disciplines, methodologies, and theories of the nature of identity and psychosis, allowing for a much-needed integration. A further strength lies in the way the analysis was carried out, with input from a multidisciplinary, collaborative team. This study has at least 3 limitations. First, it is possible that not all articles that describe a way of understanding identity change were included. As we included a broad range of terms for identity, to maintain sufficient focus, one of these identity terms had to be found in the title for its abstract to be screened. Second, we were able to include only studies available in languages that use Latin script; therefore, we may have missed other extant understandings of identity changes. Third, we used Western medical classification systems for the diagnostic inclusion criteria, thereby situating our work within Western, medicalized understandings of psychosis. There is research indicating that certain nonmedical understandings of psychotic experiences involve identity changes and loss of sense of self.^103^ To address these limitations, future research should explore identity changes in different cultures and consider nonmedical explanatory models for psychotic experiences. 

### Interpreting the Framework From a Social Identity Approach

The conceptual framework of identity changes in psychosis can be understood through the lens of the SIA, specifically the social identity model of identity change (SIMIC).^104^ According to this model, people can maintain health and well-being after important life changes to the extent that they can maintain old social group memberships or gain new social group memberships.^1,5,4–6,41–50,104^ Social groups have well-being benefits because they provide a basis for identification: with identification comes a shared sense of meaning and purpose, increased orientation to other ingroup members, and increased access to social support.^47,105–107^ Conversely, losing social group memberships can have a negative impact on mental health, as people no longer have access to these psychological resources. The SIMIC captures, and helps explain, a possible mechanism for a common understanding of identity changes: as consequences of lost roles or relationships. Considering the conceptual framework through the lens of the SIA reveals notable gaps in the way identity changes have been studied in psychosis. For example, using the SIA, research has found that having compatible (nonconflicting) identities is an important predictor of better mental health.^1 ^Changes in people’s lives may lead to seeing themselves as belonging to a new group or gaining an identity. This new identity being added (eg, being a patient) may be experienced as conflicting with a person’s existing identities. None of the reviewed articles explored the conflict between identities as a factor in identity changes that might impact people’s lives.

Second, the SIA can help us to understand how identity change may be a consequence of internalizing stigma. Research has shown that people are less likely to identify with a group that is stigmatized and that belonging to a group that is discriminated against and negatively valued can be detrimental to well-being. People cope with belonging to a negative group in different ways depending on whether the group is “achieved” (not assigned at birth) and whether it is seen as “permeable” (how possible or easy it is to leave the group).^108,109^ If a group is both achieved and permeable, its members are more likely to attempt to distance themselves from the group when it is negatively evaluated. Research in psychosis shows that people attempt to distance themselves from a schizophrenia diagnosis and often do not identify with it.^110,111^ Attempting to manage and maintain a positive sense of identity may help explain the large proportion of people with severe mental illnesses that disengage from and receive inconsistent support from services.^112–114^ Coming to see a diagnosis in a way that is positive may allow people to experience the positive effects of belonging and access more sources of support.^115^ In addition, the stigma attached to other identities may leave people vulnerable to poorer mental health and paranoia.^116,117^ For example, research among Black Britons found that identifying more with their racial identity was associated with higher levels of paranoia if their interactions with White Britons were mostly negative.^118^ This suggests that when individuals have a stigmatized identity, experiences of prejudice and discrimination related to that identity can result in mental distress.

Third, the SIA can help us to understand identity change as altered cognitive functioning. Various cognitive abilities are required for people to categorize themselves, others, and themselves in relation to others: if these cognitive abilities are changed or impaired, this will affect people’s ability to integrate different social identities into their sense of self.^34,108^

Fourth, the SIA does not suggest that all changes in identity are negative, but rather risky or pivotal times, when identities might be lost or gained. This means the SIA is compatible with understanding identity change as personal growth.

Different to the other 4 understandings, identity changes as characteristics of psychosis see identity change as “a constitutive phenotype of schizophrenia,” an integral part of psychosis rather than as a consequence of it.^12,19,119–121^ This implies a closer association between managing identity changes and treating psychosis than in the other understandings. Critics have argued against the idea that “anomalous self-experiences” are a part of illness, proposing that these may be the result of power asymmetries occurring in clinical encounters.^100,121–124^

### Implications

Although it is known that there are multiple ways of understanding identity changes in psychosis,^23,26,30^ our review provides the first conceptual framework for organizing these ideas across disparate literature. This framework offers insights into clinical and research settings by identifying and integrating potential mechanisms for interventions.

For clinicians, it is important to be aware that patients may experience identity changes as positive, negative, or neutral. Clinicians may have to adapt their therapeutic approaches to align with how patients make sense of their experiences. The understandings included in the framework could also help us identify how clinical interventions could help. Certain forms of psychotherapy have been designed to support patients with alterations in specific cognitive functions, such as metacognition, showing improvement in symptoms, social functioning, and self-experience.^125–135^ Interventions targeting stigma in the general public and internalized stigma may also support patients in having a more positive sense of self.^136–143^

For researchers, the framework can be used as a reference point for future studies, increasing transparency and allowing comparisons. Researchers may focus on one understanding from the framework or integrate different understandings of identity change. More research into identity changes as reflections of personal growth is needed, as well as research considering how power imbalances in society and in psychiatric settings might impact identity and health. In addition, the 5 understandings in this conceptual framework should be explored in first-person accounts of psychosis, as the research synthesized here is unavoidably influenced by the questions and assumptions of the researchers who wrote the included articles.

Although there are numerous existing approaches to identity change in psychosis,^23,120,135,144,145^ which provide valuable insights, we contend that there is value in adopting a social identity approach in future empirical research. The SIA provides a way of integrating all 5 understandings of identity changes in psychosis within a single overarching theory, which will allow unexplored questions to be investigated (eg, the role of conflict between identities). As paranoia is one of the most common experiences in people when first diagnosed with psychosis,^146^ research into paranoid feelings in nonclinical populations is a useful indicator for clinical research. Using the SIA, researchers have found that identifying more strongly with a group (specifically: family, nationality, and neighborhood groups) is linked to fewer paranoid feelings.^116,117,147^

## Conclusions

This systematic review and narrative synthesis presents a conceptual framework of how identity changes in psychosis are understood. The understandings stem from different theoretical frameworks that are occasionally dissonant and represent a range of patient experiences and approaches. Clinicians should be aware of the different ways of understanding identity changes and remain flexible when exploring patients’ experiences. This review provides a first step through synthesizing existing research and providing a straightforward framework of 5 understandings, which both patients and clinicians can use to help think and talk about identity changes in psychosis.

## Supplementary Material

sbaa124_suppl_Supplementary_Material_1

sbaa124_suppl_Supplementary_Material_2

sbaa124_suppl_Supplementary_Material_3
