# Supplementary material for: Understanding Identity Changes in Psychosis: A Systematic Review and Narrative Synthesis
Source: Schizophr Bull. 2020 Sep 29;47(2):309–22. doi: 10.1093/schbul/sbaa124 (PMC7965068; doi:10.1093/schbul/sbaa124)
Supplement: sbaa124_suppl_Supplementary_Material_1 [file sbaa124_suppl_supplementary_material_1.docx]

**Supplementary Material 1. Search strategy**

- 1. **MEDLINE (via PubMed)**

(((personal continuity OR "autobiographical memory" OR "identity" OR narrative continuity OR “personal narrative” OR "social perception" OR "self perception" OR "self-view" OR "self concept" OR collective identification OR "collective identity" OR group identity OR social identity OR identity))) AND ((schizophrenia OR psychosis OR psychotic OR schizo* OR voice hearer OR "hearing voices"))

MeSH Terms used:

"narration"

"identification (psychology)"

"population groups"

"social identification"

"schizophrenia"

"psychotic disorders"

“voice”

- 1. **PsycINFO**

(personal continuity OR "autobiographical memory" OR "identity" OR narrative continuity OR “personal narrative” OR "social perception" OR "self perception" OR "self-view" OR "self concept" OR collective identification OR "collective identity" OR group identity OR social identity OR identity) AND (schizophrenia OR psychosis OR psychotic OR schizo* OR voice hearer OR "hearing voices")

- 1. **Web of Science**

(personal continuity OR "autobiographical memory" OR "identity" OR narrative continuity OR “personal narrative” OR "social perception" OR "self perception" OR "self-view" OR "self concept" OR collective identification OR "collective identity" OR group identity OR social identity OR identity) AND (schizophrenia OR psychosis OR psychotic OR schizo* OR voice hearer OR "hearing voices")

- 1. **CINAHL**

(personal continuity OR "autobiographical memory" OR "identity" OR narrative continuity OR “personal narrative” OR "social perception" OR "self perception" OR "self-view" OR "self concept" OR collective identification OR "collective identity" OR group identity OR social identity OR identity) AND (schizophrenia OR psychosis OR psychotic OR schizo* OR voice hearer OR "hearing voices")

- 1. **Open Grey**

(personal continuity OR "autobiographical memory" OR "identity" OR narrative continuity OR “personal narrative” OR "social perception" OR "self perception" OR "self-view" OR "self concept" OR collective identification OR "collective identity" OR group identity OR social identity OR identity) AND (schizophrenia OR psychosis OR psychotic OR schizo* OR voice hearer OR "hearing voices")

UPDATE SEARCH 9^th^ of April 2020.
