# Supplementary material for: Understanding Identity Changes in Psychosis: A Systematic Review and Narrative Synthesis
Source: Schizophr Bull. 2020 Sep 29;47(2):309–22. doi: 10.1093/schbul/sbaa124 (PMC7965068; doi:10.1093/schbul/sbaa124)
Supplement: sbaa124_suppl_Supplementary_Material_2 [file sbaa124_suppl_supplementary_material_2.docx]

**Supplementary Material 2. Full description of modified narrative synthesis stages**

Stage 1: Developing a preliminary framework

The data from the studies were extracted and tabulated in line with the PROSPERO protocol. The methods and results sections of the included papers were used to assess the understandings of identity change. This data extraction was used to create textual descriptions of each of the included studies, brief descriptions of conceptualisation of identity change employed as the basis for a thematic analysis carried out by the coding authors (MC and PM).^29^ After initially independently coding of 25% of the texts (15 papers) to create coding frames, MC and PM discussed their coding frames and agreed on a frame that was applied to the remaining texts by MC. The coding frame was amended throughout the process of coding the texts, as new concepts emerged. Different groupings and subgroupings of studies’ were created based on common and diverging features in their understandings of identity change, and the concepts were mapped out. Thematic analysis and concept mapping were used to create a visual representation of the preliminary conceptual framework.

Stage 2: Exploring relationships within and between studies

Initial groupings of studies based on their conceptualisations of identity change were scrutinised, compared and further commonalities and differences within and across groups were underlined. The included studies were mapped onto the emergent conceptual framework and broadly similar conceptualisations of identity change were fleshed out with specific examples from studies.

Stage 3: Assessing the robustness of the conceptual framework

Throughout the process of creating and refining the conceptual framework, contradictory evidence was sought by MC to test the robustness of the synthesis. In addition, in the final stage of the narrative synthesis, ‘assessing the robustness of the conceptual framework’, we presented the conceptual framework at three meetings, to a panel of Service Users and Carers Advising on Research (SUGAR, City University of London), and to a group of 30 researchers in psychiatry (at the Unit for Social and Community Psychiatry) and to social psychologists at the Social Identity and Groups Network (SIGN, University of Queensland). The combined feedback from the different groups was the following: Firstly, to change the title of the first understanding of identity change from “a characteristic of psychosis” to something that did not describe identity change as an unavoidable part of psychosis. Secondly, it was suggested that first person accounts written by people who had experienced psychosis should be added to the analysis and the conceptual framework should be re-created combining first-person accounts and research studies. Thirdly, we were asked to clarify and put greater emphasis on the distinctions between personal and social identity changes, or inter-personal and intra-personal identity changes.

The first suggestion was considered but not taken on board as after close re-inspecting of the original studies we concluded that several studies clearly described identity change as a part of psychosis, like a symptom or a characteristic of the disorder. The second suggestion was thought to consider an equally important research question, but a different research question that could not be addressed within the scope of this single study but rather deserved its own separate analysis. The final suggested amendment was taken on board, and where the information was available in the original studies personal and social factors affecting identity change were distinguished and are summarised for the five understandings in the framework in Table 2.
