# Supplementary material for: Understanding Identity Changes in Psychosis: A Systematic Review and Narrative Synthesis
Source: Schizophr Bull. 2020 Sep 29;47(2):309–22. doi: 10.1093/schbul/sbaa124 (PMC7965068; doi:10.1093/schbul/sbaa124)
Supplement: sbaa124_suppl_Supplementary_Material_3 [file sbaa124_suppl_supplementary_material_3.docx]

**Supplementary Material 3. Table 3. Included studies’ descriptions of identity change and understandings present**

| **Author (Year)**  ***Country*** | **Title** | **Study design** | **Diagnosis (*n* with psychosis) - Setting** | **Understanding of identity change**  ***Additional details of the study if needed to understand their conceptualisation of identity change*** | **1. Characteristic of psychosis** | **2. Altered cognitive function** | **3. Internalised Stigma** | **4. Lost roles and relationships** | **5. Personal growth** |
| --- | --- | --- | --- | --- | --- | --- | --- | --- | --- |
| Allé et al. (2015)^1^  *France* | Impaired coherence of life narratives of patients with schizophrenia | Quantitative – cross-sectional study | Schizophrenia (27) – outpatient  26 controls (no diagnosis) | Identity change is understood as comprising changes on two complementary dimensions of self: the “minimal self”, “a pre-reflexive dimension of the self that supports an immediate “sense of self”” and the “narrative self”, which “corresponds to the dimension of self that is extended in time and provides a sense of continuity and coherence to the self across time” (p. 1).  *Related impairments in patients’ life narratives as to impaired executive function.* |  | **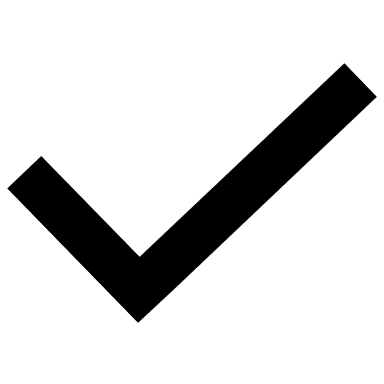** |  |  |  |
| Arnfred et al. (2015)^2^  *Denmark* | Self-Disorder and Brain Processing of Proprioception in Schizophrenia Spectrum Patients: A Re-Analysis | Quantitative – re-analysis of associations between 6 electrophysiological variables and 6 aspects of self-disorders (SD). | Schizophrenia and schizotypal personality disorder (6 and 6) - inpatient | Identity change is understood as self-disorder measured using the Examination of Anomalous Self-Experience^3^ (for description of the EASE see Parnas et al., 2005, page 14).  *The authors explored the possible association between neurocognition (abnormalities of proprioceptive evoked activity, measured using an EEG) and identity change (self-disorder). The hypothesis, based on the fact that the development of the sense of ‘self-within-an-environment’ largely relies on the establishment of sensorimotor equivalencies, was that a more disordered self (higher score on the EASE) would be associated with abnormalities in proprioceptive information processing.* | **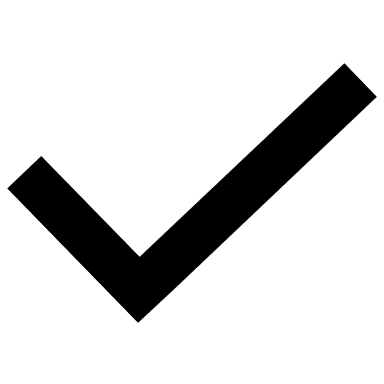** |  |  |  |  |
| Berg (1992)^4^  *United States of America* | The effects of goal-oriented sociodramatic roleplay on the locus of control, self-concept and psychiatric adjustment in schizophrenic-patients | Qualitative – controlled (TAU) pre-post design | Schizophrenia (34) - inpatient | Identity change is understood as self-concept, which is measured in interviews and discussed as either high or low.  *Changing identity with an intervention (called socio-dramatic roleplay) was hypothesised to improve overall functioning (psychiatric adjustment). No significant differences in identity or psychiatric adjustment were found.* |  |  | **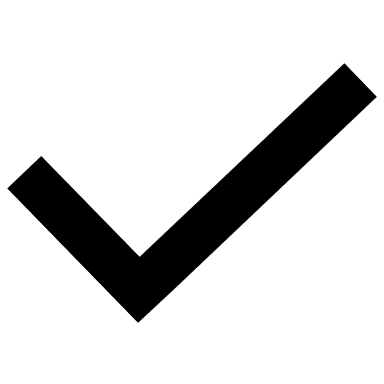** | **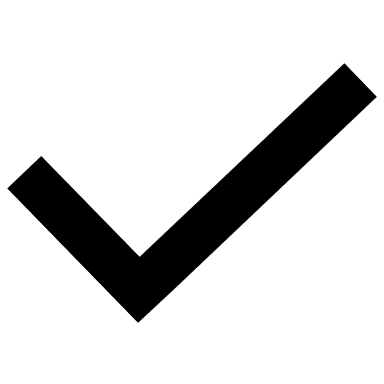** |  |
| Berkhout et al. (2019)^5^  *Canada* | Identity, Subjectivity, and Disorders of Self in Psychosis | Qualitative – repeated ethnographic in-depth interviews | First Episode Psychosis (9/17) – outpatient (9 people with psychosis, 5 clinicians, 3 family members) | Identity change in psychosis is understood as at least in part, to be the result of the power imbalances that come with a diagnosis and being treated in biomedically oriented treatment setting. The power dynamics embedded in a clinical setting are thought to be a part of the change in. The authors argue against the pure “disturbed ipseity”, or anomalous self-experience understanding of identity change in psychosis, which reduces experiences that might be made meaningful into symptoms of psychopathology. |  |  | **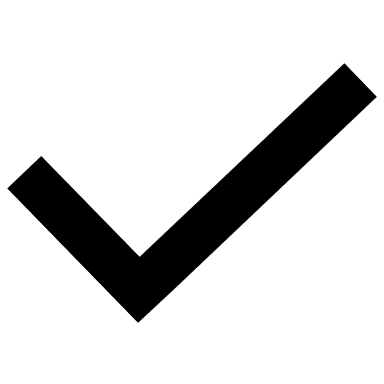** | **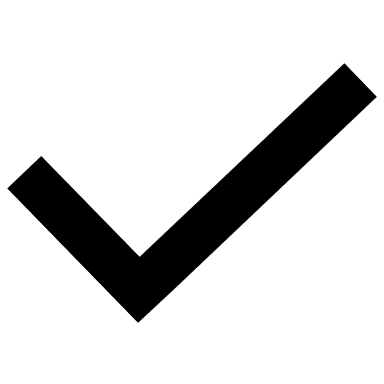** |  |
| Blairy et al. (2008)^6^  *Belgium* | Improvements in autobiographical memory in schizophrenia patients after a cognitive intervention: a preliminary study | Quantitative – Cohort pre-post design and 3-month follow-up | Chronic schizophrenia (15/27) -inpatient  12 in the control group | Identity change is understood using different constructs that are considered to be related: autobiographical memory, autonoetic awareness (“the kind of awareness experienced by normal individuals who consciously recollect personal events by reliving them mentally”) and personal identity (not defined, but a fundamental disruption in the sense of self-identity, or poor self-identity is thought to be a part of schizophrenia).  *Cognitive remediation therapy intervention was provided (it included exercises on self-definition, the memories of past, goals for the future and different potential roles, and the part all play in self-definition) and hypothesised to improve autobiographical memory and thereby depression and executive function. The intervention improved participants’ ability to remember specific events but did not have an effect on depression or executive function.* |  | **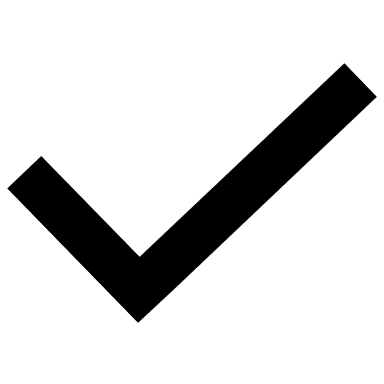** |  |  |  |
| Bourdeau et al. (2014)^7^  *Canada* | Stages of recovery in early psychosis: Associations with symptoms, function, and narrative development | Mixed methods – Longitudinal interviews (follow up at 3 and 6 months) and quantitative assessment at baseline | First Episode Psychosis (47) – outpatient | Identity change is understood in terms of recovery, using the five stages of recovery analysis grid created for the present study, and narrative development using the Scale to Assess Narrative Development (STAND, see page 7 Lysaker et al., 2003). These are the five stage of recovery that the recovery analysis grid is based on: Moratorium (hopelessness, confusion, denial, withdrawal), Awareness (regaining hope and awareness of a self capable of recovery), Preparation (identiﬁcation of values and strengths, connection with peers, development of skills), Rebuilding (forging a positive identity, taking control of one’s life, working towards personally valued goals), and Growth (resilience in the face of setbacks, living a meaningful life, positive sense of self).  Identity change is considered in terms of rebuilding following initial onset of symptoms (as can be seen in stage of the change beginning in “moratorium”). Rebuilding involves roles and connections with others, and fostering a way of seeing themselves that is positive and not about illness. |  |  | **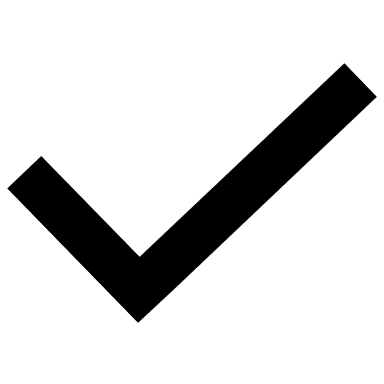** | **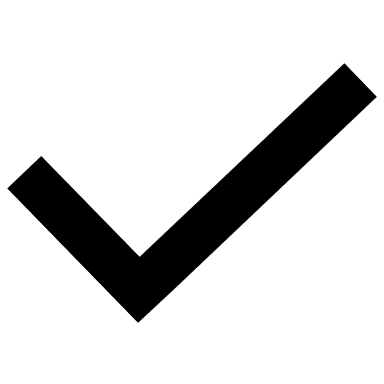** |  |
| Braehler et al. (2012)^8^  *United Kingdom* | Recovering an emerging self: exploring reflective function in recovery from adolescent-onset psychosis | Qualitative – Grounded Theory Interview | Schizophrenia Spectrum (8) - outpatient | Identity change is understood from an attachment theory perspective. Mentalisation, or reflective function is “the ability to infer one’s own and others’ mental states to predict behaviour”. Identity change is understood from an attachment theory perspective. Mentalisation, or reflective function is “the ability to infer one’s own and others’ mental states to predict behaviour”. The psychological self is seen as development of these cognitive abilities.  *Narrative (from the attachment theory interview) were categorised based on patients’ reflective function impairment, this was linked to changes in self and relationships.* |  | **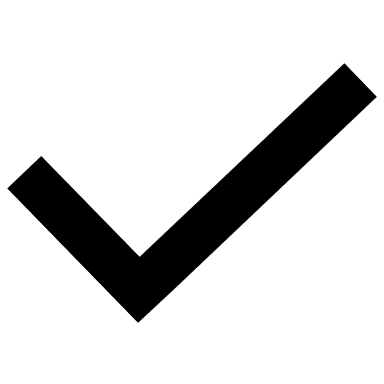** |  |  |  |
| Burnham (1961)^9^  *USA* | Identity definition and role demand in the hospital careers of schizophrenic patients | Qualitative – Case series | Schizophrenia (4) - inpatient | Identity is understood as “a person’s organised and enduring conception of himself and his place in the social scheme of things.” The patient role is defined as the “the place which others assign him”. |  |  |  | **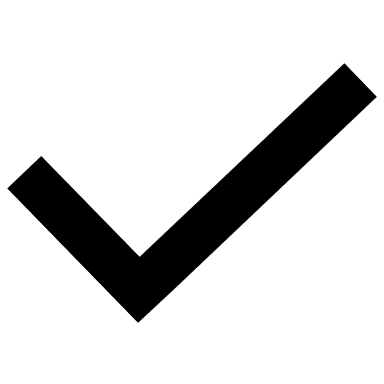** | **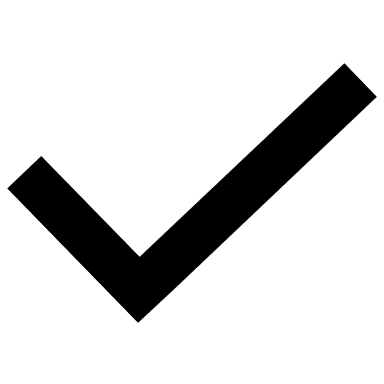** |
| Carless et al. (2008)^10^  *United Kingdom* | Narrative, identity and mental health: How men with serious mental illness re-story their lives through sport and exercise | Qualitative – ethnographic participant observation and semi-structured interviews | Schizophrenia (confirmed with authors) (11) - inpatient | Identity change is described as a social process of narrative reconstruction and meaning-making that is facilitated by having sport and exercise. |  |  |  | **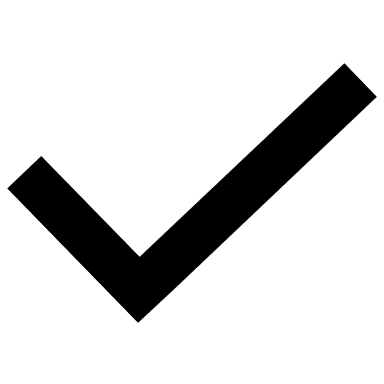** |  |
| Connell et al. (2015)^11^  *Australia* | Recovery from first-episode psychosis and recovering self: A qualitative study | Qualitative – Interpretive Phenomenological Analysis | First Episode Psychosis (20) - outpatient | Identity change understood in the tradition of the dialogical self: identity change can involve intra- and interpersonal dialogues, social roles and meaning-making.  *Two broad superordinate themes captured trends in the data: experiences of self-estrangement and self-consolidation.* |  |  | **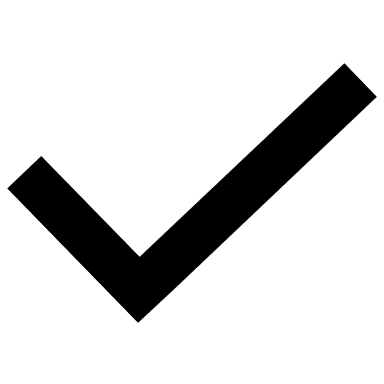** | **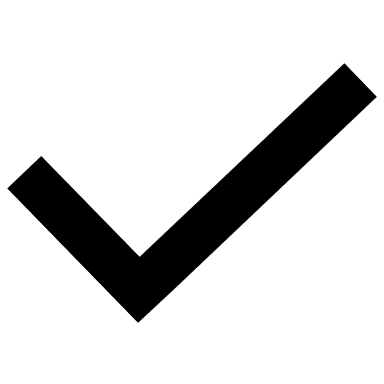** | **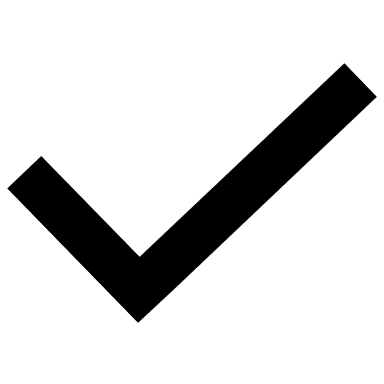** |
| Corin (1998)^12^  *Canada* | The thickness of being: intentional worlds, strategies of identity, and experience among schizophrenics | Mixed methods –Interviews and admission data | Schizophrenia (45) -outpatient | Identity change is understood as primarily related to the physical environment of the hospital and social role changes and stigma it brings with it. |  |  | **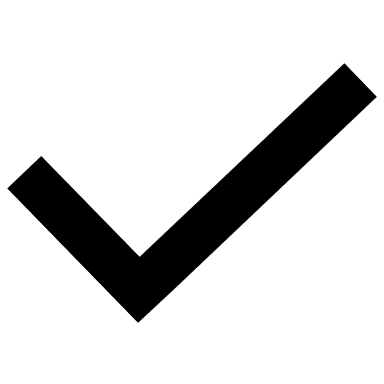** | **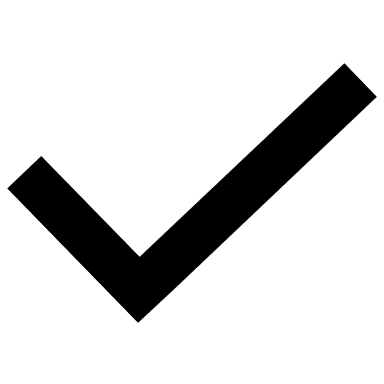** |  |
| De Vries et al. (2013)^13^  *The Netherlands* | Self-disturbance in schizophrenia: a phenomenological approach to better understand our patients | Qualitative – case reports | Schizophrenia (4) – in and outpatient | Identity change is understood as self-experience or a “disruption of the normal self-perception”.  *Describes self-disturbance in schizophrenia from case reports, and proposes these experiences are related to underlying changes in cognitive abilities resulting from psychotic illness. No cognitive measures were collected to assess cognitive ability.* |  | **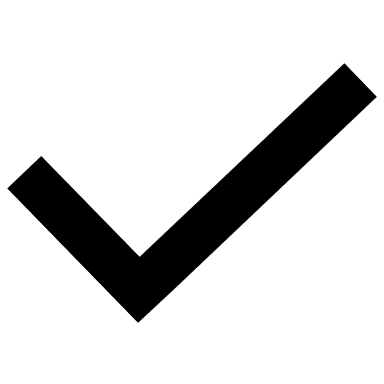** |  |  |  |
| Dunkley et al. (2015)^14^  *Australia* | Understanding the trauma of first‐episode psychosis | Qualitative –Interpretive Phenomenological Analysis longitudinal (T2: three months later) | First Episode Psychosis (10) – outpatient  (8 carers) | Identity change in psychosis is seen as resulting from a psychotic episode interrupting the formation of relationships with others and interrupting the formation of sense of self. Identity change is the “shattering of one’s beliefs about the self, others and the world” |  |  |  | **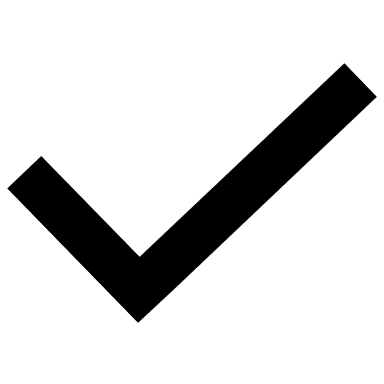** | **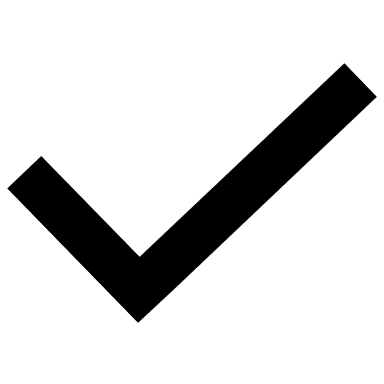** |
| Firmin et al. (2016)  *United States of America* | Veteran identity as a protective factor: A grounded theory comparison of perceptions of self, illness, and treatment among veterans and non-veterans with schizophrenia | Qualitative – Grounded Theory | Schizophrenia (46) - outpatients | Identity change is understood in social terms and is related to the roles people have and how those roles are perceive (i.e. are the positive, or negative or is stigma attached to them).  *The study investigated whether veterans and non-veterans with schizophrenia differ in their perceptions of themselves, their illness, and treatment. Findings support the theory that identifying as a veteran has several protective factors that might help shape self-perception and thereby responses to stigma, attitudes toward treatment, and hope for the future.* |  |  |  | **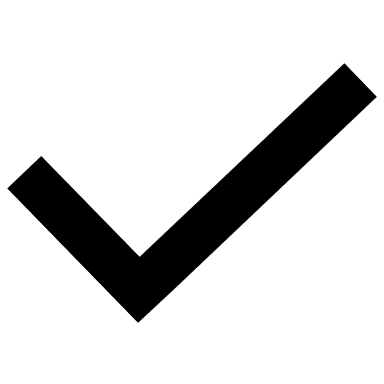** |  |
| Gomez (2006)^15^  *Spain* | The effect of a psychosocial intervention on the quality of life, symptoms and the construction of self in patients diagnosed with paranoid schizophrenia | Quantitative – Randomised controlled trial (Time 1: 12 months, Time 2: 24 months). | Schizophrenia (n=30) - outpatient | Identity change is understood using personal construct theory. The schemas and worldviews of patients are assessed using a pre-defined structure (a Repertory Grid). The self varies in the degree to which it is elaborated or un-elaborated.  Original language title : Efecto de una intervención psicosocial sobre calidad de vida, estado sintomático y construcción del sí mismo en pacientes diagnosticados de esquizofrenia paranoid [SPANISH] |  |  |  | **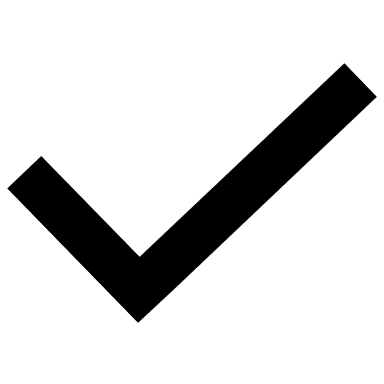** |  |
| Haug et al. (2012a)  *Norway* | The association between self-disorders and neurocognitive dysfunction in schizophrenia | Quantitative – Cross sectional survey | Schizophrenia (57) - in and outpatient | Identity is understood as self-disorder (and measured using the Examination of anomalous Self-Experience, see Parnas et al., 2005, p. 14 ^16^).  *Neurocognitive performance was measured: few associations between self-disorders and neurocognitive impairments were found.* |  | **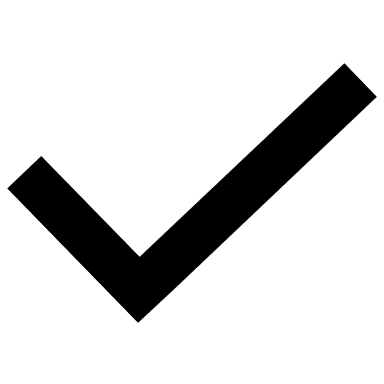** |  |  |  |
| Haug et al. (2012)^17^  *Norway* | Selective Aggregation of Self-Disorders in First-Treatment DSM-IV Schizophrenia Spectrum Disorders | Quantitative - Cross sectional survey | Schizophrenia spectrum (70/91 schizophrenia) - in and outpatient | Identity change is understood as self-disorder (measured using the Examination of anomalous Self-Experience ^16^*).  *Explored whether self-disorders (SD) can discriminate between schizophrenia spectrum disorders (schizophrenia, schizophreniform disorder, and schizoaffective disorder), bipolar psychosis (bipolar disorder I, bipolar disorder not otherwise specified) and other psychotic disorders usually classified outside of the (narrow) schizophrenia spectrum (delusional disorder and psychosis Not Otherwise Specified) in the early phase of the disorder. All models indicated that the presence of SDs (measured by the EASE total score) had a strong and statistically significant positive association with a diagnosis of schizophrenia.* | **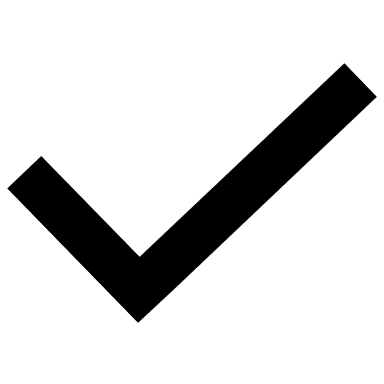** |  |  |  |  |
| Haug et al. (2012)^18^  *Norway* | The association between anomalous self-experience and suicidality in first-episode schizophrenia seems mediated by depression | Quantitative - Consecutive recruitment, cross sectional survey | Schizophrenia (49) – in and outpatient | Identity change is understood as self-disorder ( measured using the Examination of anomalous Self-Experience ^16^*).  *Investigated whether self-disorder is associated with suicidality and depression. There is a clear association between current suicidality and self-disorders, which appears to be mediated by depression.* | **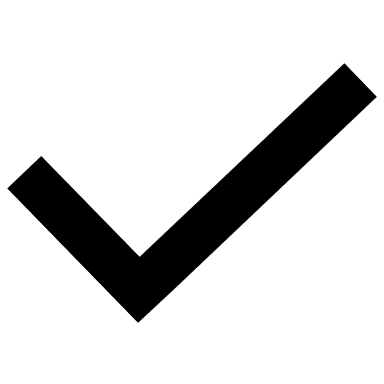** |  |  |  |  |
| Haug et al. (2017)^19^  *Norway* | High levels of anomalous self-experience are associated with longer duration of untreated psychosis | Quantitative - Cross sectional survey | Schizophrenia-spectrum – mix in and outpatient | Identity change is understood as self-disorder measured using the Examination of anomalous Self-Experience ^16^  *Investigated the relationship between anomalous self-experiences (ASEs or self-disorder) and duration of untreated psychosis (DUP). They found that high level of ASEs will have an independent association with long DUP even after correcting for other variables.* | **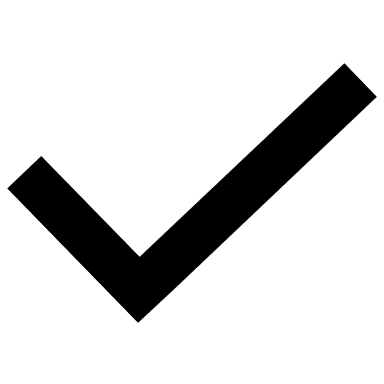** |  |  |  |  |
| Hesse et al. (2015)^20^  *Germany* | Longitudinal relations between symptoms, neurocognition, and self-concept in schizophrenia | Quantitative - longitudinal, 12 month follow-up | Schizophrenia (160) - outpatients | Identity change is understood as self-disorder (measured using the Examination of anomalous Self-Experience^16^*), a closely related construct focusing on an understanding of the self that is more social and more changeable (measured using the Frankfurt Self-Concept Scale (FSKN)^21^.  *Examined the plausibility of the scar (cognitive deficits come after the onset of symptoms) and vulnerability models (low cognitive functioning may leave people more vulnerable to developing psychosis) for the relationship between cognitive deficits, symptoms and self-concepts in people with “schizophrenic psychosis”. Findings support the scar model.* |  | **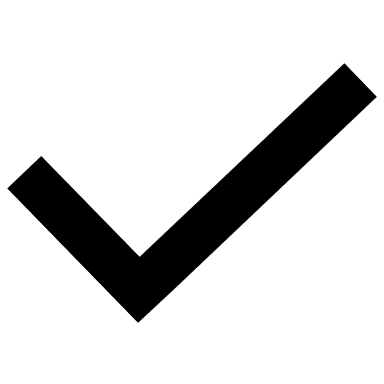** |  | **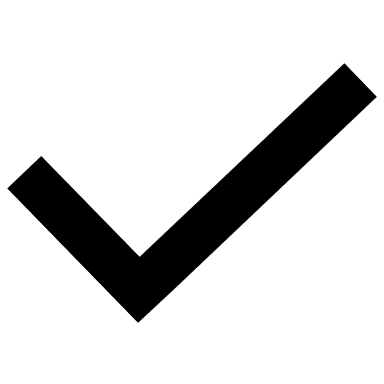** |  |
| Hesse et al. (2015b)^22^  *Germany* | The vicious cycle of family atmosphere, interpersonal self-concepts, and paranoia in schizophrenia—A longitudinal study | Quantitative – longitudinal (T2 at 12 months) | Schizophrenia (160) -outpatients | Identity change was understood as primarily social, interpersonal in nature: Interpersonal self-concept was assessed using 2 subscales of the Frankfurt Self-Concept Scales (FSKN^21^): “valued by others” and “emotions and relations to others”. |  |  |  | **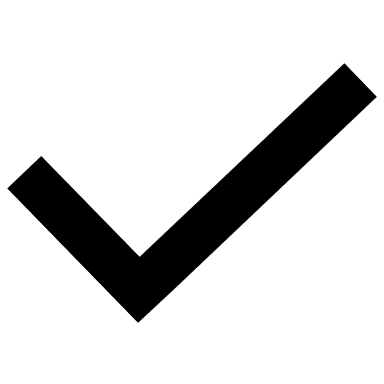** |  |
| Holm et al. (2017)^23^  *Denmark* | A decline in self-defining memories following a diagnosis of schizophrenia | Quantitative – case control (controls had no psychiatric diagnosis) | Schizophrenia (25 and 25 controls) - outpatient | Identity change is defined in different ways: individually by different participants experiences (from participants life stories, the chapters in their life stories, and their self-defining memories), and in terms of the patient role (engulfment: the degree to which people’s identities are limited to that role of being a patient). Self-defining memory is defined as a memory from your life that you remember very clearly and that still feels important to you even as you think about it.  *No difference in the mean number of self-defining memories produced by controls and patients but the temporal distribution was different – with an earlier peak (15-24 years) for patients.* |  | **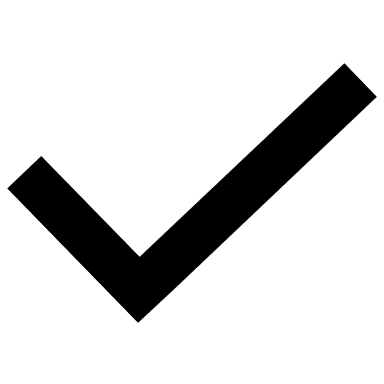** |  | **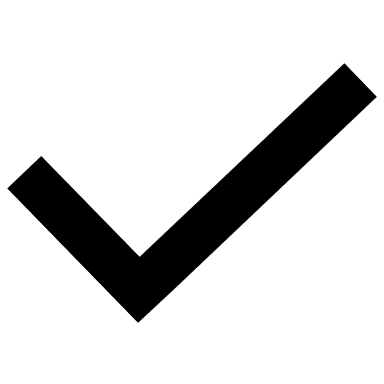** |  |
| Jarosinski (2008)^24^  *United States of America* | Exploring the experience of hallucinations from a perspective of self: surviving and persevering | Qualitative – Heideggerian and hermeneutics-guided thematic analysis | Schizophrenia and schizoaffective disorder (12) - outpatient | The understanding of identity change came from patients accounts, after being asked: “Tell me what it was like for you when you hallucinated.” How does this experience affect the way you think about yourself? • How do you see yourself? • If you did not have this experience would you think differently about yourself?  *Four themes described surviving and persevering: (a) Are they who they are? (b) a not-so-certain life; (c) finding strength in the broken places; and(d) I am still me. Although experiencing hallucinations was analogous to living with loss, participants recalled hallucinations as providing something positive in how they cared for and related to others.* |  |  | **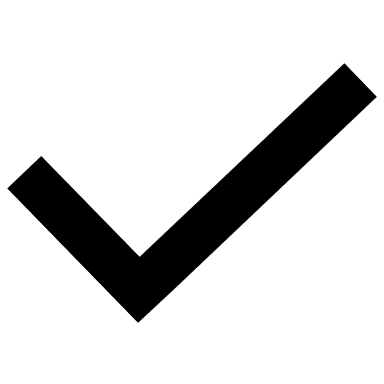** | **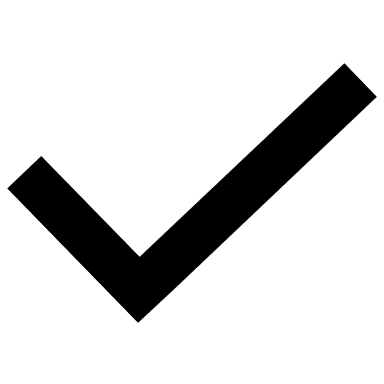** | **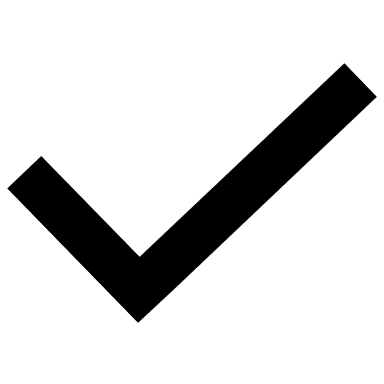** |
| Knaifel et al. (2015)^25^  *Israel* | Interplay of identities: A narrative study of self-perceptions among immigrants with severe mental illness from the former Soviet Union | Qualitative – Narrative Interview | SMI (8/12 with schizophrenia) - outpatient | Identity change was described based on participants’ experiences, focusing on change related to developing psychosis, and changes related to migration. Other identities, that are not related to illness may serve as a normalizing and positive experience.  *In the narratives participants constructed multiple identities for themselves: as bearers of Russian culture, as Soviet Jews, as immigrants, and only lastly as consumers of mental health services.* |  |  | **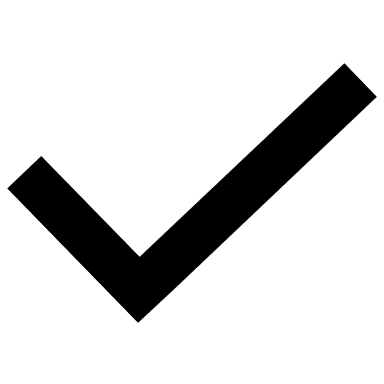** | **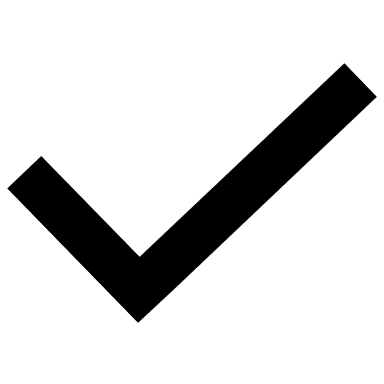** |  |
| Konsztowicz et al., (2019)^26^  *Canada* | The role of illness engulfment in the association between insight and depressive symptomatology in schizophrenia | Quantitative – Cross sectional survey | Schizophrenia or schizoaffective disorder (140) - mix of in and outpatient | Engulfment in the patient role, understood as the process by which an individual’s self-concept becomes defined solely by illness and the “patient identity becomes primary”, was assessed using the Modified Engulfment Scale. |  |  | **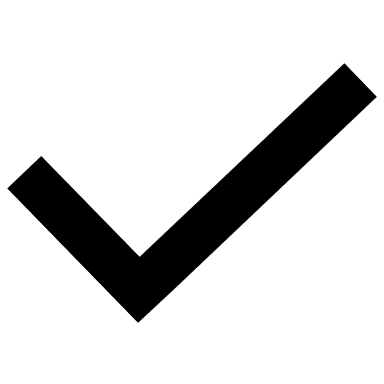** |  |  |
| Lally (1989)^27^  *United States of America* | Does being in here mean there is something wrong with me? | Mixed methods – Quantitative: Cross sectional association testing \| Qualitative: semi-structured interviews | Mixed diagnoses - Schizophrenia (32/60) – inpatient | Identity change is understood as the degree to which a person’s sense of self becomes defined solely by illness, or how engulfed they are in the patient/illness role.  *Several hypotheses were explored, for example that engulfment would be positively related to exposure to psychiatric hospitals and negatively related to the presence of multiple roles.* |  |  |  | **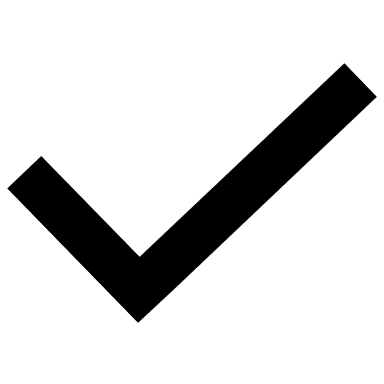** | **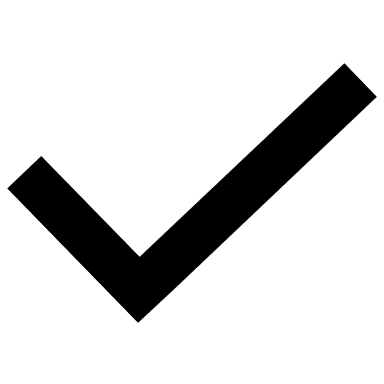** |
| Lysaker et al (2003)^28^  *United States of America* | Movement towards coherence in the psychotherapy of schizophrenia: a method for assessing narrative transformation | Quantitative – assessment of reliability and validity | Schizophrenia Spectrum (4) – outpatient | This paper describes the development of the Scale to Assess Narrative Development (STAND). The STAND is a researcher-rated scale used to measure identity change, or the “quality of self-experiences”, or “narrative transformation” of people diagnosed with schizophrenia. Identity is thought to be an important element of recovery. Based on this, this scale aims to quantify it as an indicator of how far along patients are in their recovery. The rating scale is applied by researchers to transcripts of patients’ narratives of their life and illness, usually elicited using the Indiana Psychiatric Illness Interview (IPII ^29^). The STAND is composed of four subscales: Illness Awareness (whether people describe themselves as having an illness), Alienation (how socially isolated they are), Agency (how able people feel to change or control their lives), and Social Worth (how valued they think they are by others). They each have five anchors that are rated on a 5-point Likert scale. Identity change is understood in social terms (how connected or disconnect people are from others, and valued they believe they are), and how much they feel able to make decisions and carry out actions in their life. |  |  | 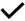 | **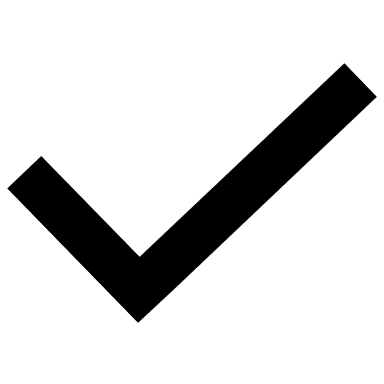** |  |
| Lysaker et al. (2006)^30^  *United States of America* | Associations of symptoms, psychosocial function and hope with qualities of self-experience in schizophrenia: comparisons of objective and subjective indicators of health | Quantitative – cross-sectional survey | Schizophrenia spectrum disorder (65) - outpatient | Identity change was understood as narrative development, measured from patients’ narratives using the Scale to Assess Narrative Development (STAND, see page 7 Lysaker et al., 2003).  *Identity change was studied in relation to symptoms of psychosis, cognitive ability, insight and to hope.* |  |  | **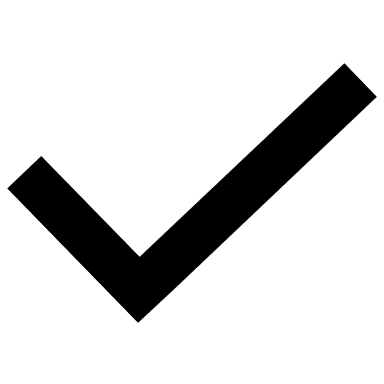** | **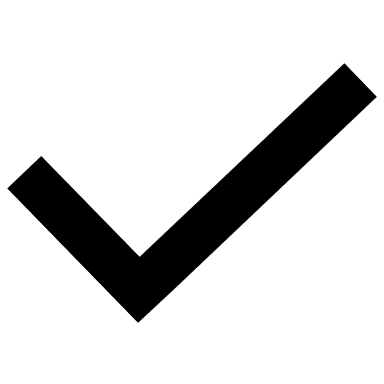** |  |
| Lysaker et al. (2006)^31^  *United States of America* | The Scale to Assess Narrative Development: association with other measures of self and readiness for recovery in schizophrenia spectrum disorders | Quantitative – cross-sectional survey | Schizophrenia, schizoaffective (34) - outpatient | Identity change was understood as narrative development, measured from patients’ narratives using the Scale to Assess Narrative Development (STAND, see page 7 Lysaker et al., 2003).  *Identity change was correlated with sense of self as valuable and readiness for positive change.* |  |  | **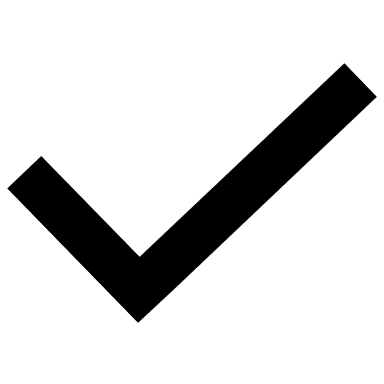** | **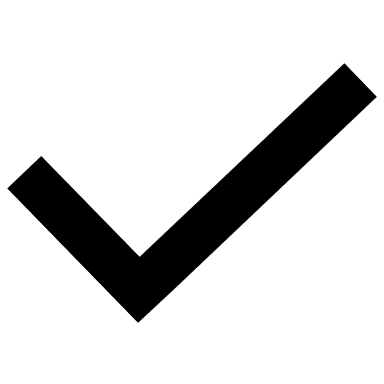** |  |
| Lysaker et al. (2008)^32^  *United States of America* | Associations of metacognition and internalized stigma with quantitative assessments of self-experience in narratives of schizophrenia | Quantitative – cross-sectional survey | Schizophrenia and schizoaffective disorder (46) - outpatient | Identity change was understood in line with, and assessed using, the Scale to Assess Narrative Development (STAND^33^, see description page 7 Lysaker et al., 2003).  *Internalised stigma and metacognitive ability were also studied. They aimed to examine whether internalized stigma and deficits in metacognition are possible barriers to the development of richer self-experience. Higher STAND ratings were significantly associated with greater ratings of metacognitive capacity (“the capacity to think about one's own thinking, for example, to the ability to name and scrutinize one's thoughts and feelings about oneself and about others”), and with less internalised stigma.* |  | **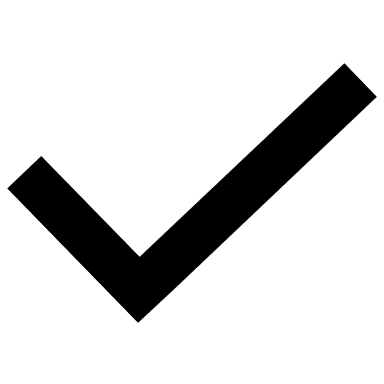** | **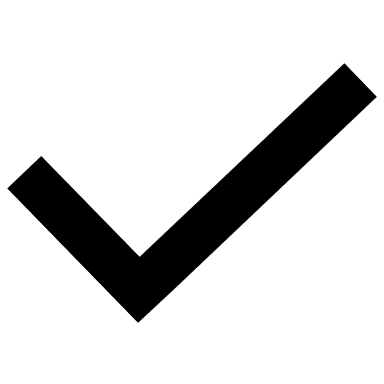** | **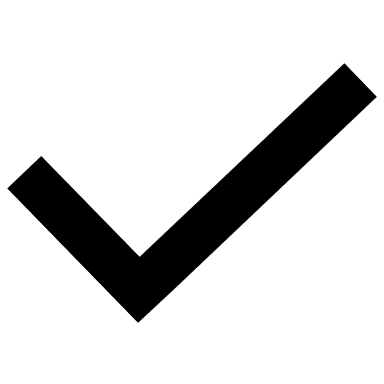** |  |
| Lysaker et al. (2012)^34^  *United States of America* | Change in self-stigma among persons with schizophrenia enrolled in rehabilitation: associations with self-esteem and positive and emotional discomfort symptoms | Quantitative – Quasi-experimental (7-month follow up) | Schizophrenia (70) - outpatient | Identity change was understood as change in self-stigma.  *Change in self-stigma was studied, before and after vocational rehabilitation. Change in self-stigma was not related to positive symptoms but was possibly related to wellbeing.* |  |  | **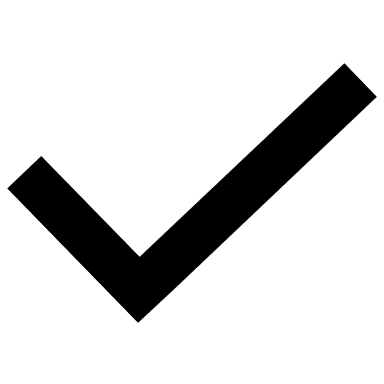** |  |  |
| Macdougall et al. (2015)^35^  *Canada* | Negative future self as a mediator in the relationship between insight and depression in psychotic disorders | Quantitative – cross sectional survey | Non-affective psychotic disorders (59% schizophrenia) (102) - outpatient | Identity change was understood forward-looking, using the construct of “possible future self”. Participants were asked to assess how much they think items such as “having friends” “, and “managing own decisions” might describe them in a few years time. This was related to self-stigma, depression and insight. as  *The study wanted to understand a finding, which is called the Stigma Paradox, that having better insight is linked to better clinical outcomes and worse well-being. They explored whether “possible future self” mediates the relationship between insight into having a mental illness and depression: i.e. is insight linked to poorer wellbeing because people who describe themselves as having a medical illness are more likely among patients of an early psychosis intervention program, independently of stigma. Negative possible self was a significant mediator of the insight–depression relationship.* |  |  | **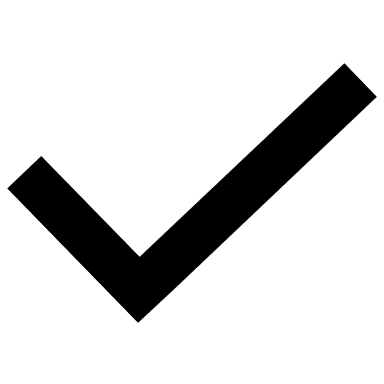** | **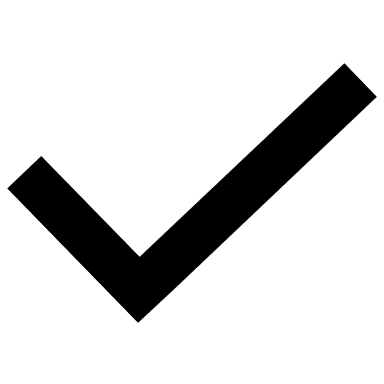** |  |
| McCay et al. (1998)^36^  *Canada* | A scale to measure the impact of a schizophrenic illness on an individual's self-concept | Quantitative – cross sectional survey | Schizophrenia (100) - outpatient | This understanding of identity change is specifically focused on change in relation to illness, i.e. how much a person’s sense of self incorporates the experience of mental illness. The Modified Engulfment Scale (MES) measures and quantifies the degree to which an individual's self-concept becomes defined solely by their illness, or, the degree to which the patient is “engulfed in the illness”. It is a 30-item measure, rated on the five-point Likert scale from “completely false” to “completely true”. The items include: "I can't do things for myself the way other people can", "I will never be the person I was before my psychiatric illness began", "Once a mental patient, always a mental patient”.  *Engulfment in the patient role correlates with hopelessness, and low self-esteem and negatively correlates with self-efficacy.* |  |  | **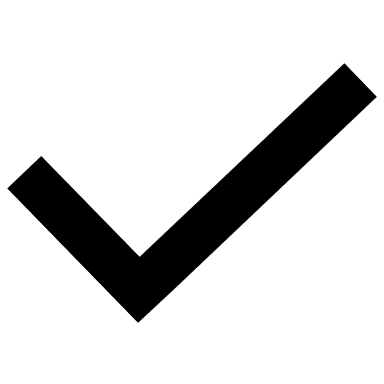** | **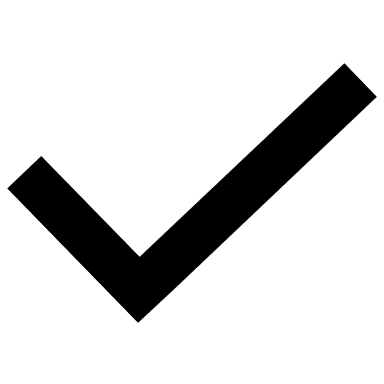** |  |
| McCay et al. (2006)^37^  *Canada* | A Group Intervention to Promote Healthy Self-Concepts and Guide Recovery in First Episode Schizophrenia: A Pilot Study | Quantitative – Non randomised controlled pre-post trial (12 week group, follow up post treatment and at 3 months post treatment) | First Episode Psychosis (FEP) (52) -outpatient | Identity change was measured using the Modified Engulfment Scale (MES, see McCay et al.,1998, page 8, for a description).  *The study tested a group intervention targeting improvement of self-concept and overall quality of life for young adults recovering from a first episode of schizophrenia. Participants in the treatment group demonstrated significant improvement in engulfment (the engulfing effects of the illness were minimised), quality of life and symptoms, while the comparison group did not change.* |  |  | **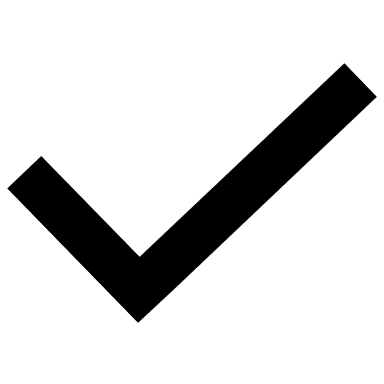** | **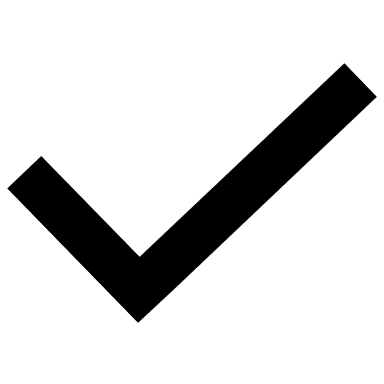** |  |
| McCay et al. (2007)^38^  *Canada* | A randomised controlled trial of a group intervention to reduce engulfment and self-stigmatisation in first episode schizophrenia | Quantitative - RCT (T2: three months) | First Episode Psychosis (FEP) (67) - outpatient | Modified Engulfment Scale was used to measure identity change (see McCay et al., 1998, page 8, for description).  *The study evaluated a group intervention designed to promote healthy self-concepts by reducing self-stigmatisation and engulfment among young adults recovering from first episode schizophrenia. The treatment group significantly improved on engulfment, hope, and quality of life compared with the control.* |  |  | **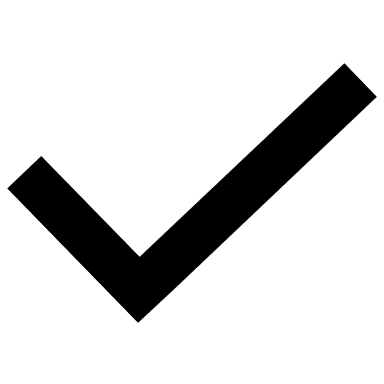** | **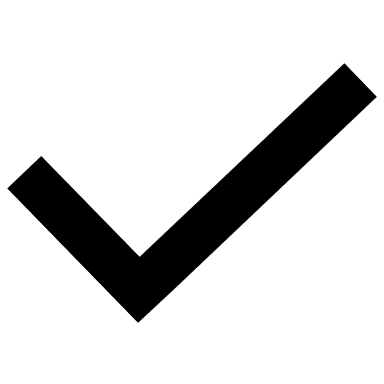** |  |
| McEvoy et al. (1982)^39^  *Papua New Guinea* | Sociocultural identity and psychogenic psychosis | Qualitative – case series | “Psychogenic psychosis” (3) – in and outpatient | Description of individual cases of socio-cultural stress precipitating psychosis. “The cases described here in which social-cultural stress apparently precipitated acute psychosis are taken from the first author’s experience in the psychiatric service of a Papua New Guinean general hospital”. The author argues that psychosis is related to socio-cultural factors and that exposure to two or more very different cultures is an identity conflict that could aggravate or initiate psychosis. In all three cases are different identity conflicts related to migration and/or growing up between cultures. The content of the psychotic episode experienced is culture-related and changing the cultural setting cured the psychosis. |  |  | **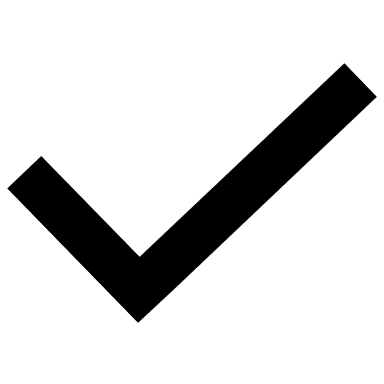** | **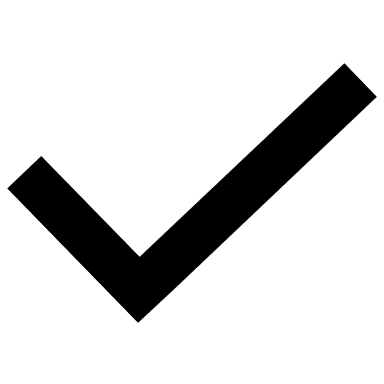** |  |
| McGuire et al. (2015)^40^  *United States of America* | Altered self-experience and goal setting in severe mental illness | Qualitative - Interviews | Veterans with various diagnosis including 66% schizophrenia (21) - outpatient | Identity change is explored qualitatively in interviews, informed by dialogical self-theory. The different categories of self-experience from dialogical self-theory were used to categorise patients (from their narratives). There are four categories of self-experience are: Open (considered optimal; “different aspects of the self, or self-positions, interanimate or give significance to one another through their interaction or dialogue …a fluid sense of personal identity is made possible), Barren (“lack of meaningful interaction between self-facets”), Cacophonous (“involves all self-facets simultaneously vying for space within the dialogue”) and Monological (“one self-facet dominates the dialogue, to the exclusion of other self-facets”). |  |  |  | **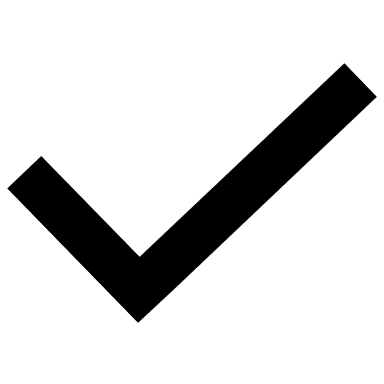** |  |
| Meehan et al. (2008)^41^  *Ireland* | Self-construction in schizophrenia: a discourse analysis | Qualitative - Discourse Analysis | Schizophrenia (8) - outpatient | Identity change was described as related to roles, and the perception of those roles (stigma associated with psychosis). |  |  | **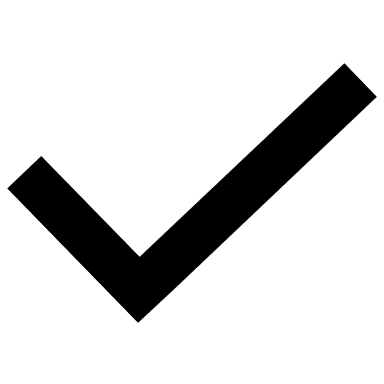** | **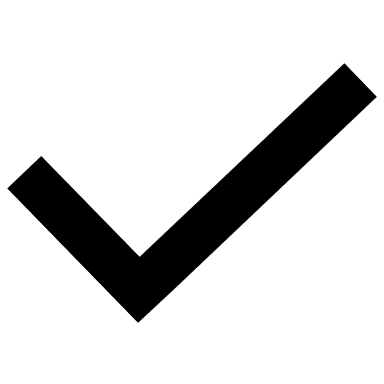** |  |
| Moe et al. (2013)^42^  *United States of America* | Schizophrenia and the sense of self | Quantitative – Cross sectional analysis based on narrative self-descriptions | Schizophrenia (N = 50/90) - outpatients | Identity change was assessed based on patients’ narratives.  *Participants were asked: “Describe yourself as fully as you can” and the transcripts were analysed using select scales from the Assessment of Self-Descriptions^43^. The 9 sub-scales most relevant to the measurement of self (as conceptualised for the present study) chosen were: to assess aspects of relatedness (articulation of relatedness, quality of relatedness, and level of relatedness), sense of agency (negative-positive self-regard, self-critical, striving/ambitious, and level of self-definition), modes of self-description (substantiality), and integration of the self (differentiation/integration).* | **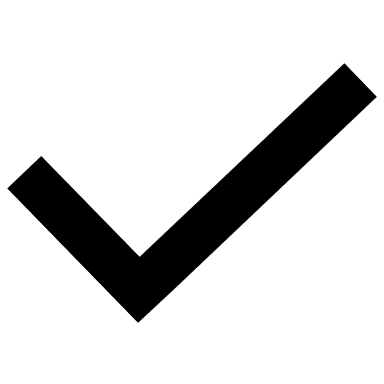** |  |  | **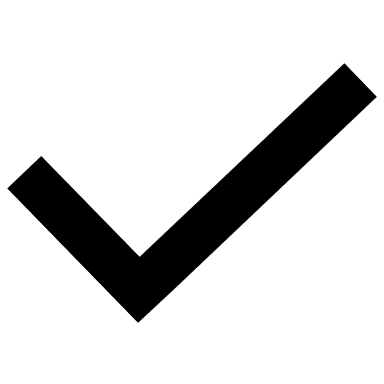** |  |
| Monville (2018)  *Belgium* | Delusion, Self and Neuroleptics. A phenomenological approach to the effects of neuroleptics | Qualitative – Retrospective case series | Schizophrenia (6) – outpatient | The understanding of identity change used combines self-disorder as it is understood according to Schneider (i.e. as a first rank symptom of schizophrenia), and Sass’s understating of self-disorder (i.e. as falling into three domains (diminished self, hyper reflexivity, loss of stability in the self in observing different objects). These understandings of identity change are explored in six people, and links are made between the content of delusions and change in identity.  Original language title : Délire, Self et Neuroleptique : Approche phénoménologique de l’effet des neuroleptiques. [FRENCH] | 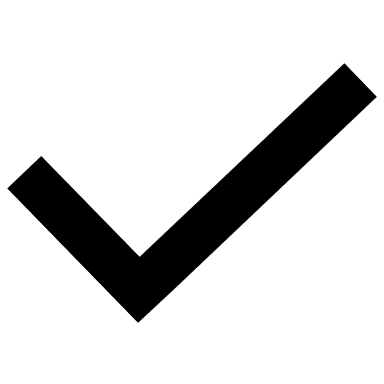 |  | 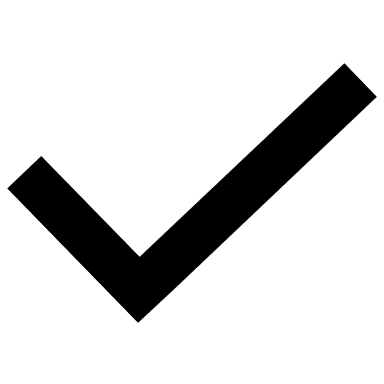 |  |  |
| Nordgaard et al. (2017)^44^  *Denmark* | Self‑disorders in schizophrenia‑spectrum disorders: a 5‑year follow‑up study | Quantitative – longitudinal survey. T2: 5 years | Schizophrenia Spectrum (48) – inpatient at baseline, unspecified at follow up | Identity change is understood as self-disorder, measured using the Examination of Anomalous Self-Experiences (Examination of anomalous Self-Experience; Parnas et al., 2005, page 10, for description) | **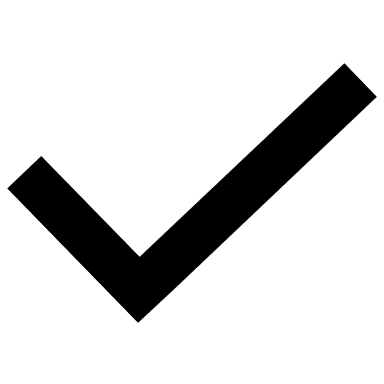** |  |  |  |  |
| Nordgaard et al. (2014)^45^  *Denmark* | Self-disorders and the Schizophrenia Spectrum: A Study of 100 First Hospital Admissions | Quantitative – cross sectional survey | Schizophrenia Spectrum (100, 68 psychosis and schizotypal) - inpatient | Identity change understood as self-disorder and measured using the Examination of Anomalous Self-Experiences to assess Self-Disorder (EASE; Parnas et al. 2005).  IQ was assessed. No correlation was found between IQ and EASE. | **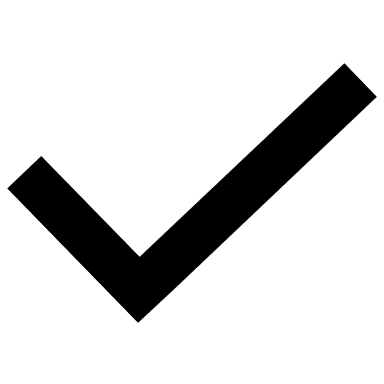** | **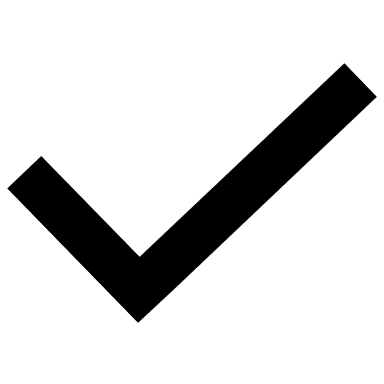** |  |  |  |
| Parnas et al. (2003)^16^  *Denmark* | Phenomenology of anomalous self-experience in early schizophrenia | Qualitative - Case series | Schizophrenia – mix in and outpatient (17) | Interviews and clinical assessments explore self-disturbances or self-disorders. “Self-disorders may turn out to be potentially useful as a psychopathological organizer of the schizophrenia spectrum disorders”. | **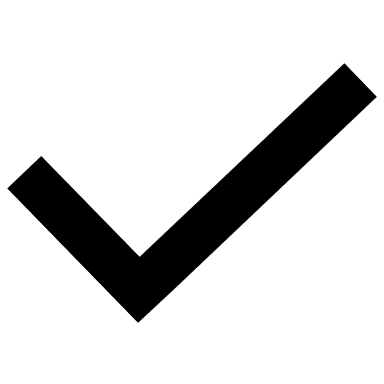** |  | **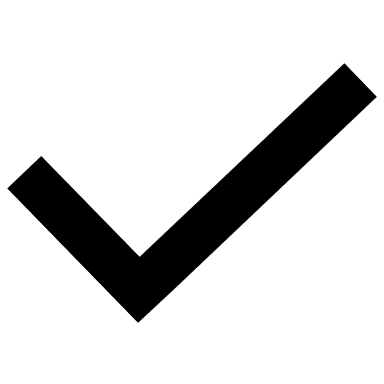** |  |  |
| Parnas et al. (2014)^46^  *Denmark* | Premorbid self-disorders and lifetime diagnosis in the schizophrenia spectrum: a prospective high-risk study | Quantitative – Longitudinal study (Follow up approximately 5, 10 and 25 years after the initial assessment) | Schizophrenia spectrum (68) - outpatient | Identity change understood as self-disorder – measured using a subset of items from the Minnesota Multiphasic Personality Inventory (MMPI), which is a quantitative, self-rated measure, based on their similarity to EASE content domain. “Our criterion would be to select any MMPI item statement that, if spontaneously presented as a complaint by the subject in an EASE interview, would motivate the EASE interviewer to explore it in-depth as a potential token of self-disorder”.  For a description of the EASE (Examination of Anomalous Self-Experience, Parnas et al. 2003, p. 11). | **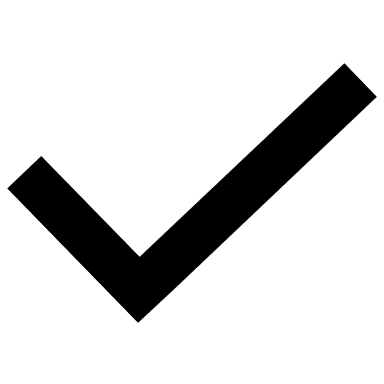** |  |  |  |  |
| Perez-Corrales et al. (2019)^47^  *Spain* | ‘Being normal’ and self-identity: the experience of volunteering in individuals with severe mental disorders-a qualitative study | Qualitative – interviews and researcher field notes | Schizophrenia Spectrum (23) - outpatient | Semi-structured interviews about how volunteering influences their life and sense of self. Two main themes 1) the impact volunteering has on sense of self through acquiring a new role, through feeling pride and accomplishment in themselves and being given responsibility, and feeling valued by others and having covered the helpful and 2) normalising effect of a socially acceptable role (being a volunteer) in a non-clinical setting that brings distance from the identity of a person who is ill. |  |  | **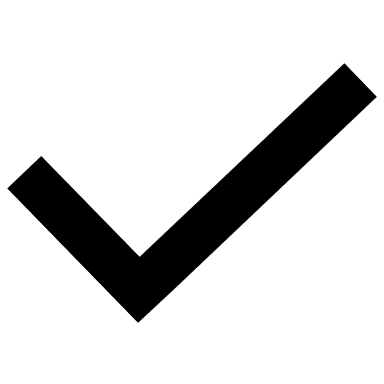** | **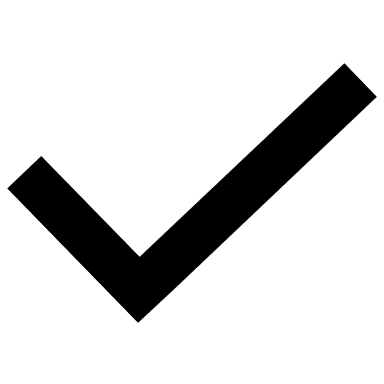** |  |
| Raballo et al. (2018)^48^  *Norwa*y | The Self in the Spectrum: A Closer Look at the Temporal Stability of Self-Disorders in Schizophrenia | Quantitative – secondary analysis of longitudinal data, T2: 5 years later | Schizophrenia spectrum, first admission (48) - inpatient | Identity change was understood as self-disorder, measured with the EASE. EASE-measured self-disorder (Examination of Anomalous Self Experience^3^, see Parnas 2003, page 11 for description).  *Self-disorder shows a high temporal persistence over a 5-year period.* | **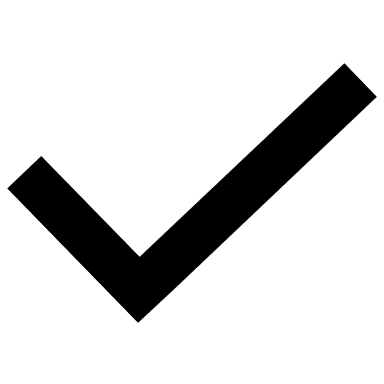** |  |  |  |  |
| Raballo et al. (2012)^49^  *Norwa*y | Examination of anomalous self-experience: initial study of the structure of self-disorders in schizophrenia spectrum | Quantitative – cross sectional survey | Schizophrenia or schizotypal (27/36) - inpatient | Identity change was understood as self-disorder, measured using the Examination of Anomalous Self Experience (EASE)^50^.  *Explored self-disorders and their association to symptoms and duration of illness in admissions to hospital suspected of having a psychotic disorder. The diagnostic subgroups (schizophrenia, schizotypal disorder, and non-schizophrenia spectrum) were found to differ in terms of self-disorders.* | **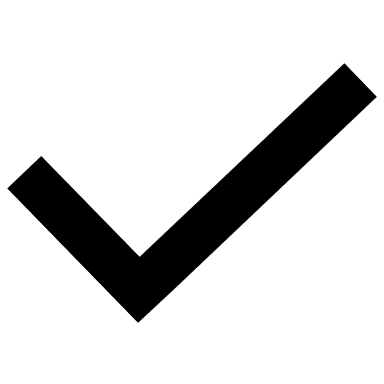** |  |  |  |  |
| Rohricht et al. (2004)^51^  *United Kingdom* | Ego-pathology and common symptom factors in schizophrenia | Quantitative – longitudinal T1: 3 days after admission, T2: 2 weeks after T1 | Paranoid schizophrenia (60) - inpatient | Identity change was understood as self-disorder and measured using the ego pathology inventory (EPP; see Scharfetter, page 14 for a description).  *The study explores whether ego-pathology scores overlap with or are distinct from common psychopathology, i.e., general, positive, and negative symptom factors. Explores if ego-pathology scores could be a useful predictor of for short-term treatment outcome.* | **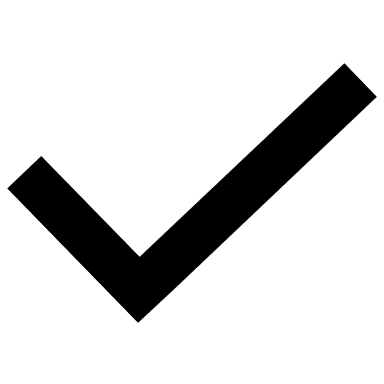** |  |  |  |  |
| Rohricht et al. (2009A)^52^  *United Kingdom* | The association of religiosity, spirituality, and ethnic background with ego-pathology in acute schizophrenia | Quantitative – Exploratory cross-sectional survey | Acute Paranoid Schizophrenia (42) - inpatient | Identity change was understood as a negative change that was a sign of psychotic illness. Two methods used to measure identity change 1) the Ego Pathology Inventory (EPP; see Scharfetter on page 14 for a description); 2) semi-structured interviews exploring spirituality, religiosity and “What do the terms “self” and “ego” mean to you?” “What do you think is the most important quality of everybody’s ego?”.  *Quality of life was correlated with ego-pathology and not with religiosity nor spirituality. The results are taken as evidence for the notion of schizophrenia as severe ego-disorder and that “disturbed ego-pathology” are “a core psychopathological phenomena"* | **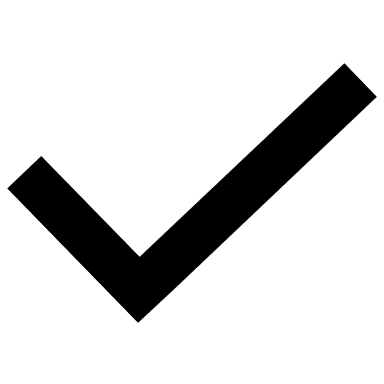** |  |  |  |  |
| Rohricht et al. (2009B)^53^  *United Kingdom* | Ego-pathology, body experience, and body psychotherapy in chronic schizophrenia | Quantitative – Pilot Randomised Controlled Trial | Schizophrenia at least 2 episodes of acute psychotic symptoms (24) - outpatient | Identity change was understood using the framework provided by the Ego Pathology Inventory (EPP, see Scharfetter on page 14 for a description).  *The aims of the study were to explore whether (1) Ego-psychopathology and (2) Body experiences, may change during body psychotherapy.* | **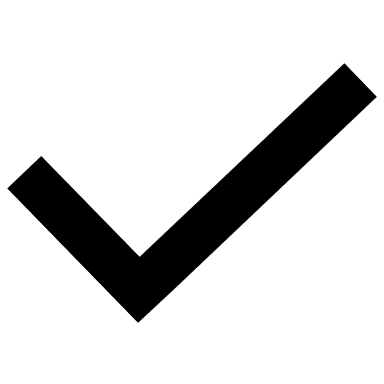** |  |  |  |  |
| Romano et al. (2010)^54^  *Canada* | Reshaping an enduring sense of self: the process of recovery from a first episode of schizophrenia | Qualitative – Grounded Theory, two time points | First Episode Schizophrenia (10) - outpatient | Identity change is understood from in-depth interviews as related to cognitive processes, roles and was experienced by some participants as personal growth and transformation. “Participants’ enduring sense of self were reshaped rather than reconstructed throughout the process”  *Lead to a theory of the process of recovery that is comprised of phases: ‘Who they were prior to the illness’, ‘Lives interrupted: Encountering the illness’, ‘Engaging in services and supports’, ‘Re-engaging in life’, ‘Envisioning the future’; and the core category, ‘Re-shaping an enduring sense of self’, that occurred throughout all phases.* |  | **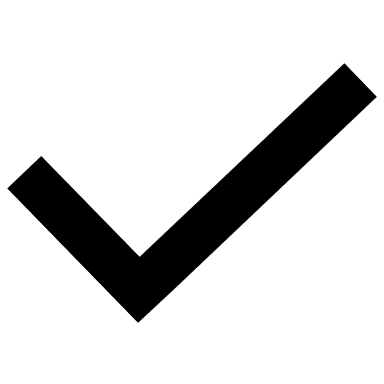** |  | **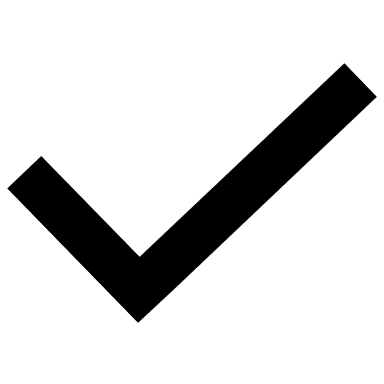** | **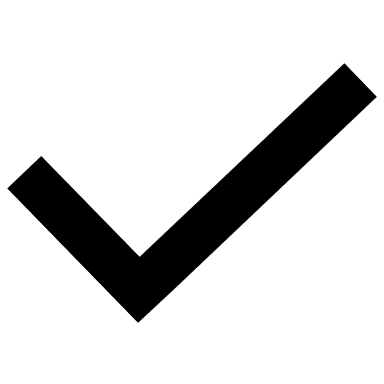** |
| Rusch et al. (2009)^55^  *United States of America* | Self-stigma, group identification, perceived legitimacy of discrimination and mental health service use | Quantitative – longitudinal 6 month follow up | Mixed diagnoses, 54% schizophrenia (39/75) – outpatient | Identity is understood using two constructs: self-stigma and group identification. (with the group of people with mental illness).  *Examines the impact of self-stigma and stigma-related cognitions on service use.* |  |  | **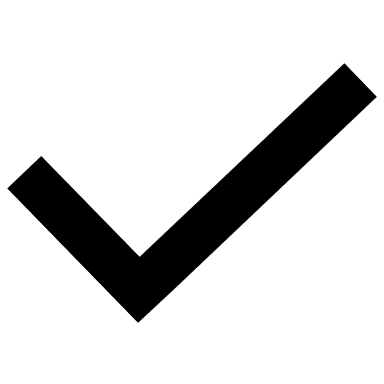** |  |  |
| Saavedra (2009)^56^  *Spain* | Schizophrenia, narrative and change: Andalusian care homes as novel sociocultural context | Qualitative – narrative interviews and quantitative analyses of frequencies of utterances. T2: 2 years | Schizophrenia (10) – inpatient in a care home (50% long-stay, 50% short stay). | Identity change is described based on open autobiographical interviews: the narratives of participants relate the construction of social identity and recovery.  *Differences in length of stay are used to explain how the important social network a care home can be in influencing identity change. Twenty percent more mentions of social relationships in long-stay than short-stay patients.* |  |  |  | **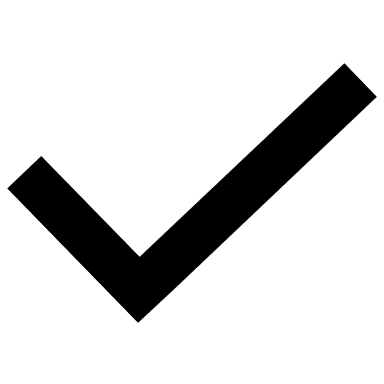** |  |
| Salerno et al. (2001)^57^  *United States of America* | Hope, power, and perception of self in individuals recovering from schizophrenia: A Rogerian science perspective | Quantitative – cross sectional survey | Schizophrenia (107) – outpatient | Identity change is understood as how “unitary the perception one has of oneself is” which is “manifested by an individual’s awareness of the infinite wholeness of the human field”. The scale, called the Human Field Image Metaphor Scale, is based on a Rogerian perspective of self and “uses metaphor as a means of promoting unitary field expression”. “A higher score on the HFIMS represents expressions of a strong sense of integrity and an expanded perception of potential; whereas, a lower score represents a greater sense of isolation and a diminished perception of potential.” |  |  |  |  | **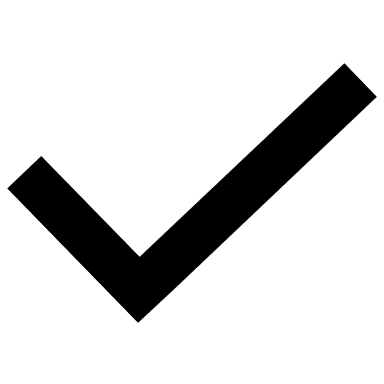** |
| Scharfetter (1981)^58^  *Germany* | Ego-psychopathology: the concept and its empirical evaluation | Quantitative – case-histories of people diagnosed with schizophrenia | Schizophrenia (60) – mix in and outpatient | Identity change is understood as ego-pathology, a construct closely related to self-disorder. Ego-pathology is “a phenomenological construct of ego-consciousness, subdivided into 5 aspects (vitality, activity, consistency, demarcation and identity).” The Ego Pathology Inventory captures certainty in sense of self, attitudes to life and death (self-non-existence), sense of personal ability, issues of control and change in sense of internal vs external control, and body-oriented changes. | **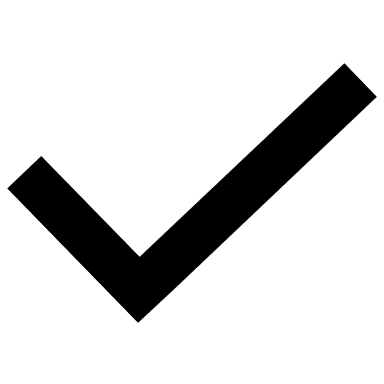** |  |  |  |  |
| Shea (2010)^59^  *United States of America* | Coming back normal: the process of self-recovery in those with schizophrenia | Qualitative – Grounded theory, repeated interviews | Schizophrenia (10) – outpatient | Identity change is described from people’s experiences: recovery is seen as a multi-stage process of self-identity reconstruction.  *A theory of the stages that people go through is developed from the interviews, the last of which is “coming back normal” and involves consolidating different parts of your identity, integrating the past and future self. Identity change is spoken of as an achievement. Other people’s views and roles and relationships are seen as important in the journey to self.* |  |  | **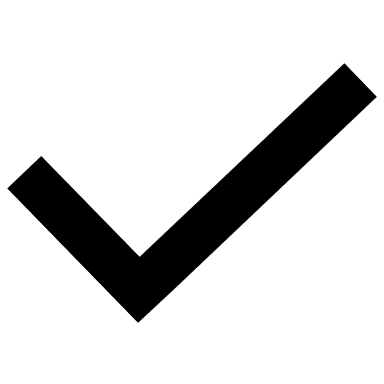** | **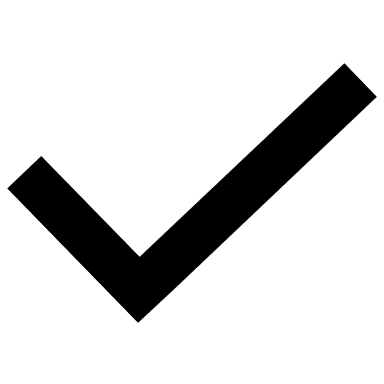** |  |
| Sugawara (2018)^60^  *Japan* | The self-concept of person with chronic schizophrenia in Japan | Qualitative – content analysis, repeated interviews | Schizophrenia (53) – mix in and outpatient | Identity change was understood using predefined understandings of self-concept consisting of six categories: Present Cognition and Definition of the Self, Feeling Towards and Evaluation of the Self, The Self That Is Seen by Others, The Past Self-Image, The Self-Image About Possibility and the Future and How I Should Be and the Ideal Self. |  |  | **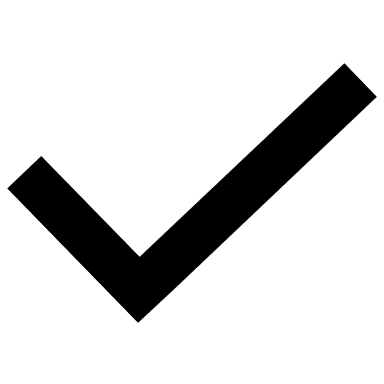** | **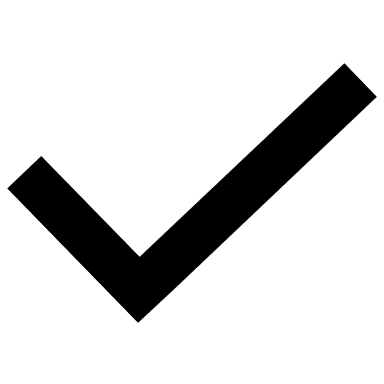** |  |
| Weinberg et al. (2012)^61^  *Israel* | Role of the Self in Schizophrenia: A Multidimensional Examination of Short-Term Outcomes | Quantitative – Longitudinal, (follow up of self-esteem daily for six days and full follow up at six weeks) | Schizophrenia-spectrum (101) – outpatient | Identity change is understood as change in “self-concept clarity” defined as “the extent to which the contents of an individual’s self-concept (perceived personal attributes) are clearly and confidently defined, internally consistent, and temporally stable”. Changes in three other constructs were also used to capture self-construct: self-esteem, self-esteem instability, and the perception of the self as ill. |  |  | **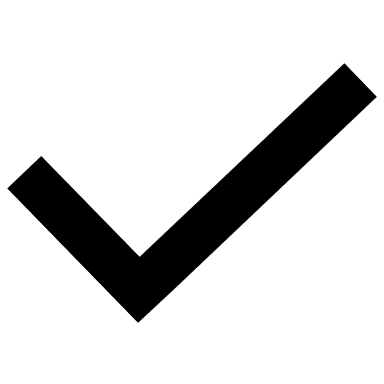** | **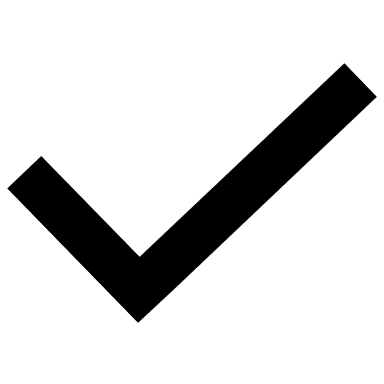** |  |
| Wright et al. (2018)^62^  *United Kingdom* | Self-defining Memories Predict Engagement in Structured Activity in First Episode Psychosis, Independent of Neurocognition and Metacognition | Quantitative – Cross sectional survey | First Episode Psychosis (71/128) (47 controls) | Self-defining, or autobiographical, memories (SDM), are the personal memories that are the ingredients for the life story and part of narrative identity. Identity change is thought to result from a complex set of processes related so symptoms, to neurocognitive changes and integration of SDMs. Integration is the capacity to incorporate memories into self-knowledge and narrative. Integration may enable people to interpret what happens in their lives and to themselves, helping define who they are as a person. Psychotic symptoms are thought to disrupt this ability. |  | **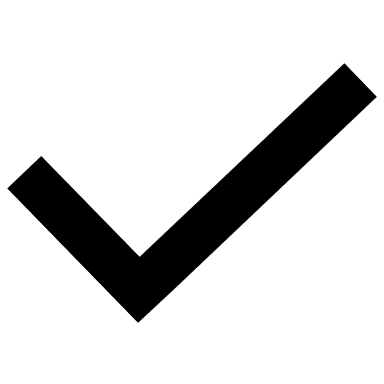** |  |  |  |

| *Examination of Anomalous Self-Experience (EASE) – Parnas et al., 2005^63^* |
| --- |
| The Examination of Anomalous Self-Experience (EASE) measures anomalous self-experience or self-disorder. The EASE is rated using 57 items based on an interview. The items cover a broad range of factors that fit into 5 categories: cognition, and thought disturbances (such as thought block, or thought pressure), self-awareness and presence (this includes items such as patients’ “inseparable sense of self and sense of immersion in the world” and a “sense of being alienated from oneself”), changes in bodily experiences (such as experiencing unusual bodily sensations, or changes in their own face’s appearance), demarcation/transitivism (whether they experience themselves as separate or merged with the world), and existential reorientation (important changes in values, and or change in their perception of themselves). Within these types of self-disorder there are different sub-types. The EASE produces a quantified measure of self-disorder. |

**References**

**1.** Alle MC, Potheegadoo J, Kober C, Schneider P, Coutelle R, Habermas T, Danion JM, Berna F. Impaired coherence of life narratives of patients with schizophrenia. *Scientific reports* Aug 10 2015;5:12934.

**2.** Arnfred SM, Raballo A, Morup M, Parnas J. Self-disorder and brain processing of proprioception in schizophrenia spectrum patients: a re-analysis. *Psychopathology* 2015;48(1):60-64.

**3.** Parnas J, Handest P, Jansson L, Sæbye D. Anomalous Subjective Experience among First-Admitted Schizophrenia Spectrum Patients: Empirical Investigation. *Psychopathology* 2005;38(5):259-267.

**4.** Berg GN. THE EFFECTS OF GOAL-ORIENTED SOCIODRAMATIC ROLEPLAY ON THE LOCUS OF CONTROL, SELF-CONCEPT AND PSYCHIATRIC ADJUSTMENT IN SCHIZOPHRENIC-PATIENTS. *International Journal of Psychology* Jun-Aug 1992;27(3-4):462-462.

**5.** Berkhout SG, Zaheer J, Remington G. Identity, subjectivity, and disorders of self in psychosis. *Culture, Medicine, and Psychiatry: An International Journal of Cross-Cultural Health Research* 2019.

**6.** Blairy S, Neumann A, Nutthals F, Pierret L, Collet D, Philippot P. Improvements in autobiographical memory in schizophrenia patients after a cognitive intervention: a preliminary study. *Psychopathology* 2008;41(6):388-396.

**7.** Bourdeau G, Lecomte T, Lysaker PH. Stages of recovery in early psychosis: Associations with symptoms, function, and narrative development. *Psychology and Psychotherapy: Theory, Research and Practice* 2015;88(2):127-142.

**8.** Braehler C, Schwannauer M. Recovering an emerging self: exploring reflective function in recovery from adolescent-onset psychosis. *Psychology and psychotherapy* Mar 2012;85(1):48-67.

**9.** Burnham DL. Identity definition and role demand in the hospital careers of schizophrenic patients. *Psychiatry* May 1961;24(2)Suppl:96-122.

**10.** Carless D, Douglas K. Narrative, identity and mental health: How men with serious mental illness re-story their lives through sport and exercise. *Psychology of Sport and Exercise* Sep 2008;9(5):576-594.

**11.** Connell M, Schweitzer R, King R. Recovery from first-episode psychosis and recovering self: A qualitative study. *Psychiatric rehabilitation journal* Dec 2015;38(4):359-364.

**12.** Corin E. The thickness of being: intentional worlds, strategies of identity, and experience among schizophrenics. *Psychiatry* Summer 1998;61(2):133-146.

**13.** de Vries R, Heering HD, Postmes L, Goedhart S, Sno HN, de Haan L. Self-disturbance in schizophrenia: a phenomenological approach to better understand our patients. *The primary care companion for CNS disorders* 2013;15(1).

**14.** Dunkley JE, Bates GW, Findlay BM. Understanding the trauma of first‐episode psychosis. *Early intervention in psychiatry* 2015;9(3):211-220.

**15.** Gómez AM, Botella L. Efecto de una intervención psicosocial sobre calidad de vida, estado sintomático y construcción del sí mismo en pacientes diagnosticados de esquizofrenia paranoide. = The effect of a psychosocial intervention on the quality of life, symptoms and the construction of self in patients diagnosed with paranoid schizophrenia. *International Journal of Clinical and Health Psychology* 2007;7(2):349-367.

**16.** Parnas J, Handest P. Phenomenology of anomalous self-experience in early schizophrenia. *Comprehensive psychiatry* Mar-Apr 2003;44(2):121-134.

**17.** Haug E, Lien L, Raballo A, Bratlien U, Oie M, Andreassen OA, Melle I, Moller P. Selective aggregation of self-disorders in first-treatment DSM-IV schizophrenia spectrum disorders. *The Journal of nervous and mental disease* Jul 2012;200(7):632-636.

**18.** Haug E, Melle I, Andreassen OA, Raballo A, Bratlien U, Oie M, Lien L, Moller P. The association between anomalous self-experience and suicidality in first-episode schizophrenia seems mediated by depression. *Comprehensive psychiatry* Jul 2012;53(5):456-460.

**19.** Haug E, Oie M, Andreassen OA, Bratlien U, Nelson B, Melle I, Moller P. High levels of anomalous self-experience are associated with longer duration of untreated psychosis. *Early intervention in psychiatry* Apr 2017;11(2):133-138.

**20.** Hesse K, Kriston L, Wittorf A, Herrlich J, Wölwer W, Klingberg S. Longitudinal relations between symptoms, neurocognition, and self-concept in schizophrenia. *Frontiers in psychology* 2015;6.

**21.** Deusinger IM. *Die Frankfurter Selbstkonzeptskalen (FSKN)*. Göttingen: Hogrefe; 1986.

**22.** Hesse K, Kriston L, Mehl S, Wittorf A, Wiedemann W, Wölwer W, Klingberg S. The vicious cycle of family atmosphere, interpersonal self-concepts, and paranoia in schizophrenia—A longitudinal study. *Schizophrenia bulletin* 2015;41(6):1403-1412.

**23.** Holm T, Pillemer DB, Bliksted V, Thomsen DK. A decline in self-defining memories following a diagnosis of schizophrenia. *Comprehensive psychiatry* Jul 2017;76:18-25.

**24.** Jarosinski JM. Exploring the experience of hallucinations from a perspective of self: surviving and persevering. *Journal of the American Psychiatric Nurses Association* 2008;14(5):353-362.

**25.** Knaifel E, Mirsky J. Interplay of identities: A narrative study of self-perceptions among immigrants with severe mental illness from the former Soviet Union. *Transcultural psychiatry* Feb 2015;52(1):74-95.

**26.** Konsztowicz S, Lepage M. The role of illness engulfment in the association between insight and depressive symptomatology in schizophrenia. *Journal of psychiatric research* 2019;111:1-7.

**27.** Lally SJ. "Does being in here mean there is something wrong with me"? *Schizophrenia bulletin* 1989;15(2):253-265.

**28.** Lysaker PH, Wickett AM, Campbell K, Buck KD. Movement towards coherence in the psychotherapy of schizophrenia: a method for assessing narrative transformation. *The Journal of nervous and mental disease* Aug 2003;191(8):538-541.

**29.** Lysaker PH, Clements CA, Plascak-Hallberg CD, Knipscheer SJ, Wright DE. Insight and personal narratives of illness in schizophrenia. *Psychiatry: Interpersonal and Biological Processes* 2002;65(3):197-206.

**30.** Lysaker PH, Buck KD, Hammoud K, Taylor AC, Roe D. Associations of symptoms, psychosocial function and hope with qualities of self-experience in schizophrenia: comparisons of objective and subjective indicators of health. *Schizophrenia research* Feb 28 2006;82(2-3):241-249.

**31.** Lysaker PH, Taylor A, Miller A, Beattie N, Strasburger A, Davis LW. The Scale to Assess Narrative Development: association with other measures of self and readiness for recovery in schizophrenia spectrum disorders. *The Journal of nervous and mental disease* Mar 2006;194(3):223-225.

**32.** Lysaker PH, Buck KD, Taylor AC, Roe D. Associations of metacognition and internalized stigma with quantitative assessments of self-experience in narratives of schizophrenia. *Psychiatry research* Jan 15 2008;157(1-3):31-38.

**33.** Lysaker PH, Lancaster RS, Lysaker JT. Narrative transformation as an outcome in the psychotherapy of schizophrenia. *Psychology and psychotherapy* Sep 2003;76(Pt 3):285-299.

**34.** Lysaker PH, Roe D, Ringer J, Gilmore EM, Yanos PT. Change in self-stigma among persons with schizophrenia enrolled in rehabilitation: associations with self-esteem and positive and emotional discomfort symptoms. *Psychological services* Aug 2012;9(3):240-247.

**35.** MacDougall AG, Vandermeer MR, Norman RM. Negative future self as a mediator in the relationship between insight and depression in psychotic disorders. *Schizophrenia research* Jun 2015;165(1):66-69.

**36.** McCay EA, Seeman MV. A scale to measure the impact of a schizophrenic illness on an individual's self-concept. *Archives of psychiatric nursing* Feb 1998;12(1):41-49.

**37.** McCay E, Beanlands H, Leszcz M, Goering P, Seeman MV, Ryan K, Johnston N, Vishnevsky T. A group intervention to promote healthy self-concepts and guide recovery in first episode schizophrenia: A pilot study. *Psychiatric rehabilitation journal* Fall 2006;30(2):105-111.

**38.** McCay E, Beanlands H, Zipursky R, et al. A randomised controlled trial of a group intervention to reduce engulfment and self-stigmatisation in first episode schizophrenia. *AeJAMH (Australian e-Journal for the Advancement of Mental Health)* 2007;6(3):1-9.

**39.** McEvoy JP, Abernethy V. Sociocultural identity and psychogenic psychosis. *Papua and New Guinea medical journal* Mar 1982;25(1):55-59.

**40.** McGuire AB, Lysaker PH, Wasmuth S. Altered self-experience and goal setting in severe mental illness. *American Journal of Psychiatric Rehabilitation* 2015;18(4):333-362.

**41.** Meehan T, MacLachlan M. Self construction in schizophrenia: a discourse analysis. *Psychology and psychotherapy* Jun 2008;81(Pt 2):131-142.

**42.** Moe AM, Docherty NM. Schizophrenia and the sense of self. *Schizophrenia bulletin* Jan 2014;40(1):161-168.

**43.** Blatt S, Bers S, Schaffer C. The assessment of self descriptions. *Unpublished manuscript (research manual), Yale University, New Haven, Conn* 1993.

**44.** Nordgaard J, Nilsson LS, Sæbye D, Parnas J. Self-disorders in schizophrenia-spectrum disorders: a 5-year follow-up study. *Eur Arch Psychiatry Clin Neurosci.* Vol 268; 2018:713-718.

**45.** Nordgaard J, Parnas J. Self-disorders and the Schizophrenia Spectrum: A Study of 100 First Hospital Admissions. *Schizophrenia bulletin* Nov 2014;40(6):1300-1307.

**46.** Parnas J, Carter J, Nordgaard J. Premorbid self-disorders and lifetime diagnosis in the schizophrenia spectrum: a prospective high-risk study. *Early intervention in psychiatry* Feb 2016;10(1):45-53.

**47.** Pérez-Corrales J, Pérez-de-Heredia-Torres M, Martínez-Piedrola R, Sánchez-Camarero C, Parás-Bravo P, Palacios-Ceña D. ‘Being normal’ and self-identity: the experience of volunteering in individuals with severe mental disorders—a qualitative study. *BMJ open* 2019;9(3):e025363.

**48.** Raballo A, Preti A. The Self in the Spectrum: A Closer Look at the Temporal Stability of Self-Disorders in Schizophrenia. *Psychopathology* 2018;51(4):285-289.

**49.** Raballo A, Parnas J. Examination of anomalous self-experience: initial study of the structure of self-disorders in schizophrenia spectrum. *The Journal of nervous and mental disease* Jul 2012;200(7):577-583.

**50.** Parnas J, Moller P, Kircher T, Thalbitzer J, Jansson L, Handest P, Zahavi D. EASE: Examination of Anomalous Self-Experience. *Psychopathology* Sep-Oct 2005;38(5):236-258.

**51.** Rohricht F, Priebe S. Ego-pathology and common symptom factors in schizophrenia. *The Journal of nervous and mental disease* Jun 2004;192(6):446-449.

**52.** Rohricht F, Basdekis-Jozsa R, Sidhu J, Mukhtar A, Suzuki I, Priebe S. The association of religiosity, spirituality, and ethnic background with ego-pathology in acute schizophrenia. *Mental Health, Religion & Culture* 2009;12(6):515-526.

**53.** Rohricht F, Papadopoulos N, Suzuki I, Priebe S. Ego-pathology, body experience, and body psychotherapy in chronic schizophrenia. *Psychology and psychotherapy* Mar 2009;82(Pt 1):19-30.

**54.** Romano DM, McCay E, Goering P, Boydell K, Zipursky R. Reshaping an enduring sense of self: the process of recovery from a first episode of schizophrenia. *Early intervention in psychiatry* Aug 2010;4(3):243-250.

**55.** Rüsch N, Corrigan Pw, Wassel A, Michaels P, Larson JE, Olschewski M, Wilkniss S, Batia K. Self-stigma, group identification, perceived legitimacy of discrimination and mental health service use. *The British Journal of Psychiatry* 2009;195(6):551-552.

**56.** Saavedra J. Schizophrenia, narrative and change: Andalusian care homes as novel sociocultural context. *Culture, medicine and psychiatry* 2009;33(2):163-184.

**57.** Salerno EM. *Hope, power, and perception of self in individuals recovering from schizophrenia: A Rogerian science perspective (Martha E. Rogers)*. US, ProQuest Information & Learning; 2001.

**58.** Scharfetter C. Ego-psychopathology: The concept and its empirical evaluation. *Psychological medicine* 1981;11(2):273-280.

**59.** Shea JM. Coming back normal: the process of self-recovery in those with schizophrenia. *Journal of the American Psychiatric Nurses Association* Jan 2010;16(1):43-51.

**60.** Sugawara H, Mori C. The self-concept of person with chronic schizophrenia in Japan. *Neuropsychopharmacol Rep* Sep 2018;38(3):124-132.

**61.** Weinberg D, Shahar G, Noyman G, Davidson L, McGlashan TH, Fennig S. Role of the Self in Schizophrenia: A Multidimensional Examination of Short-Term Outcomes. *Psychiatry: Interpersonal & Biological Processes* Fall2012 2012;75(3):285-297.

**62.** Wright AC, Davies G, Fowler D, Greenwood KE. Self-defining memories predict engagement in structured activity in first episode psychosis, independent of neurocognition and metacognition. *Schizophrenia bulletin* 2019;45(5):1081-1091.

**63.** Parnas J, Handest P, Jansson L, Saebye D. Anomalous subjective experience among first-admitted schizophrenia spectrum patients: empirical investigation. *Psychopathology* Sep-Oct 2005;38(5):259-267.
